# Self- vs. External-Regulation Behavior Scale^TM^ in different psychological contexts: A validation study

**DOI:** 10.3389/fpsyg.2022.922633

**Published:** 2022-10-26

**Authors:** Jesús de la Fuente, Mónica Pachón-Basallo, José Manuel Martínez-Vicente, Francisco Javier Peralta-Sánchez, Angélica Garzón-Umerenkova, Paul Sander

**Affiliations:** ^1^School of Education and Psychology, University of Navarra, Pamplona, Spain; ^2^School of Psychology, University of Almería, Almería, Spain; ^3^Department of Psychology, Fundación Universitaria Konrad Lorenz, Bogotá, Colombia; ^4^Department of Psychology, Teesside University, Middlesbrough, United Kingdom

**Keywords:** self-regulated behavior/context, non-regulated behavior/context, dys-regulated behavior/context, validation, self- vs. external-regulation theory

## Abstract

The *self- vs. external-regulation behavior theory*, SR-ER Theory (2021) model has postulated the Self-Regulation /Non or De-Regulation/Dys-regulation (SR-NR-DR) continuum in the person and in their context. The model also generates a behavioral heuristic that allows us to predict and explain the variability of other dependent behavioral variables in a range of scenarios (clinical, educational, health and technology contexts). Consequently, the objective of this study was to validate the different scales prepared on the basis of the theory presented. A total of 469 students voluntarily completed at different times the five questionnaires presented, to give a total of 1,385 completed questionnaires. Using an *ex post facto* design, descriptive, correlational, confirmatory factorial analysis (CFA), reliability, and concurrent validity analyses were carried out. The scales were analyzed individually and as a whole. The results showed the acceptable structure of scale and consistent levels of reliability. The five levels generated by the SR-NR-DR (personal and contextual) combinatory heuristic that arises from the theoretical model determined significant differences in the levels of the variables analyzed for each psychological context. We discuss the theoretical implications and the implications for the assessment and improvement of the behaviors analyzed in function of the personal and contextual regulation levels evaluated.

## Introduction

Classical theoretical psychological models of human self-regulatory behavior (*Self-Regulation, SR*) have been fertile ground for work on defining, conceptualizing, evaluating, and creating strategies to improve self-regulation (Carver and Scheier, 1981; Mischel, 1981). From the seminal work of Bandura (1991) in his Social Cognitive Theory in which he described the construct of Self-Regulation until today there has been an avalanche of research. Searching for the term *self-regulation* in Google Scholar produced 1.95 million articles, an indication of the level of research interest in this area of study. Further, a search for *self-regulation and health* yielded 1.45 million articles and another for *self-regulation and education* gave 1.44 million articles.

There is copious support from both research findings and theoretical works for the importance of self-regulation as a psychological construct and the need to measure self-regulation (Pandey et al., 2018; Solé-Ferrer et al., 2019). Work in classical self-regulation theory has thus far focused on determining the contribution of self-regulation to the variability of studied behaviors. However, like other concepts in Psychology, the concept of self-regulation behavior is continuously developing as researchers endeavor to explain and better adapt to the reality studied. Our research group identified that this research approach left out of account psychological phenomena whose relationship with different levels or types of self-regulation has been insufficiently considered and did not adequately explore the extent to which context is predictive of self-regulatory behavior. That realization raised a number of questions that gave rise to this line of research (new theory of Self-Regulatory Behavior). Does self-regulatory behavior carry with it different meanings or levels that have not thus far been sufficiently examined? Can self-regulation be seen as a characteristic of the subject alone? Alternatively, should we also assume that context (depending on its nature) can promote or not promote self-regulation and may operate in the same way in terms of predicting such behavior? These open questions, raised by our research team, gave rise to the new theoretical model that supports this work (de la Fuente et al., 2022a). Finally, we concluded that it was necessary to create the new scales presented here. For this reason, the objectives of this manuscript are two: (1) to synthetically show the underlying theoretical construct; there are other recent works that do it more precisely (de la Fuente et al., 2022a), (2) present the structure and initial validation process of the Scales that allow it to be evaluated.

### The *classical theory* of self-regulation

*Self-Regulation* (SR) is a construct of personality (Mithaug, 1993; Boekaerts et al., 1999; Hoyle, 2010) that describes the capacity of people to exercise planning, monitoring, and evaluation of their own behavior (Karoly, 1993; Brown, 1998; Vohs and Baumeister, 2016; Koopmann et al., 2019; Robson et al., 2020). The abundant prior research has shown SR’s positive association with factors such as personal adjustment (Mithaug, 1993; Wrosch et al., 2003) and its associations with aspects of personality: positive with conscientiousness and negative with neuroticism (Guido et al., 2015; de la Fuente et al., 2020b). An association has also been shown with behavioral adjustment in academic performance (Becker et al., 2014; Blair and Raver, 2015; Akfırat et al., 2016; Panadero, 2017; Bernardo et al., 2019; Alonso-Tapia et al., 2020). The classical understanding of the construct can be found in the work of Pervin (1988). Early notions of SR, based as they were on a molecular psychological analysis (de la Fuente et al., 2020b), had three common *principles*:

1. SR is a variable of the subject and is determined by other variables or factors particular to the subject, such as aspects of personality and metacognition (Hoyle, 2010; Malanchini et al., 2019; Jacqueline et al., 2020; Valikhani et al., 2020; Vega et al., 2020).
2. Contextual factors are of secondary importance and do not have a significant role in explaining the variability of behavioral regulation in the individual or its level, either in general or specifically in relation to education and health.
3. Individuals have higher or lower levels of SR; there are no defined categories of SR, merely degrees of SR.

### The *new vision* of self-regulated vs. externally regulated behavior theory (SR–ER)

**Figure 1:**
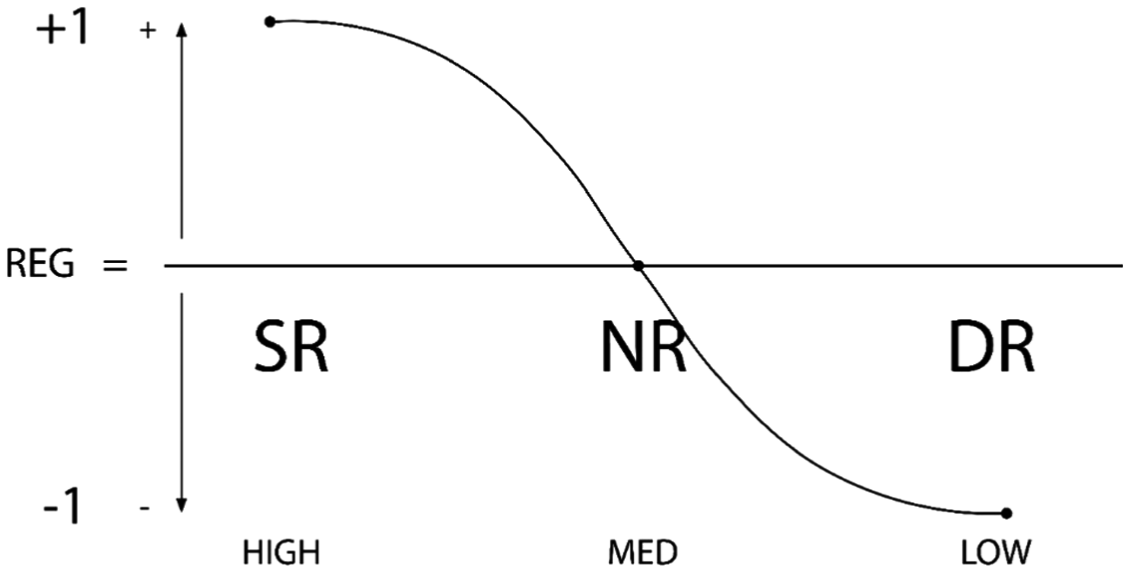
Graphic representation of the types of regulation: SR (Self-Regulation), NR (Non-Regulation or De-Regulation) and DR (Dys-Regulation). The degree of regulation (high-medium-low) is plotted on the x axis and the y axis shows positivity/negativity (+1, 0, −1). The curved line shows the possible types of regulatory stages of a person and also the possible directionality of behavioral change.

This *Self- vs. External- Regulated Behavior Theory*, or *SR vs ER Theory* model (de la Fuente, 2021b; de la Fuente et al., 2022a) has emerged to specify and expand the previous explanatory model, based exclusively on Self-Regulation (SR) variable (for a review, focused on the Educational Psychology context, please, see: de la Fuente, 2017). Through a molar analysis, this new model seeks to analyze the interaction between the regulatory characteristics of the person and the regulatory characteristics of their context (de la Fuente et al., 2020a). The SR-ER model is based on three *principles and hypothesis*:

*Principle and Hypothesis 1: Types of Behavioral Regulation*. Self-Regulation is a personal variable, which can be gradual, that is, levels or typologies can be established:

1. *Self-Regulation Behavior Type* (SR): It is the action of self-regulation (planning, self-control, and self-assessment) or internal regulation of the three levels of behavior: thoughts, emotions, and actions. It is considered an adaptative and positively proactive behavioral level (SR = +1).
2. *Non-Regulation or De-regulation Behavior Type* (NR): It can be considered as the action of ceasing to regulate or moving to a behavioral state of non-regulation of thoughts, emotions, and actions. It is considered a reactive or neutral behavioral level in positive and negative proactivity (SR = 0).
3. *Dys-Regulation Behavior Type* (DR): It refers to being unable to control behavior (thoughts, emotions, actions) in the way most people can. Before a situation. It supposes an excessive level of response (hyper-response or behavioral excesses) or negligible (under-response or behavioral deficits) that would characterize this type of behavior level. It is considered an adaptative and negative proactive behavioral level (SR = −1). See Figure 1.

In this case, the concept of SR is assumed from Zimmerman’s previous model (Zimmerman, 2000; Zimmerman and Labuhn, 2012), but the types of non-regulatory behavior are incorporated, such as the absence of regulation and dysregulatory behavior such as malfunction of regulation. In the biological field, the concept of biological dysregulation has been coined to define the malfunction of a biological system (Goldman et al., 2006; Gouin, 2011; Carbone, 2020); consequently, it is possible to coin the term behavioral dysregulation this term in the psychological field. Previous behavioral research has also assumed it to define a maladjusted psychological or behavioral level (Beauchaine and Crowell, 2020; Forkus et al., 2020). The American Psychological Association (APA) defines dysregulation as “any excessive or otherwise poorly managed mechanism or response^1^”. In the field of psychology, a commonly discussed type of dysregulation is that of emotion dysregulation, which can negatively impact our well-being. Such is the human capacity for behavioral regulation that the individual can carry out SR, NR, and DR behaviors. These types of self-regulation are then associated with the three possible levels of SR (high-medium-low) whereby positive SR describes the presence of self-regulation whilst there are two levels for absence of regulation. SR and NR can therefore be expected to be negatively associated, whilst NR and DR are positively associated, such that NR is the intermediate or prior step toward DR.

**Figure 2:**
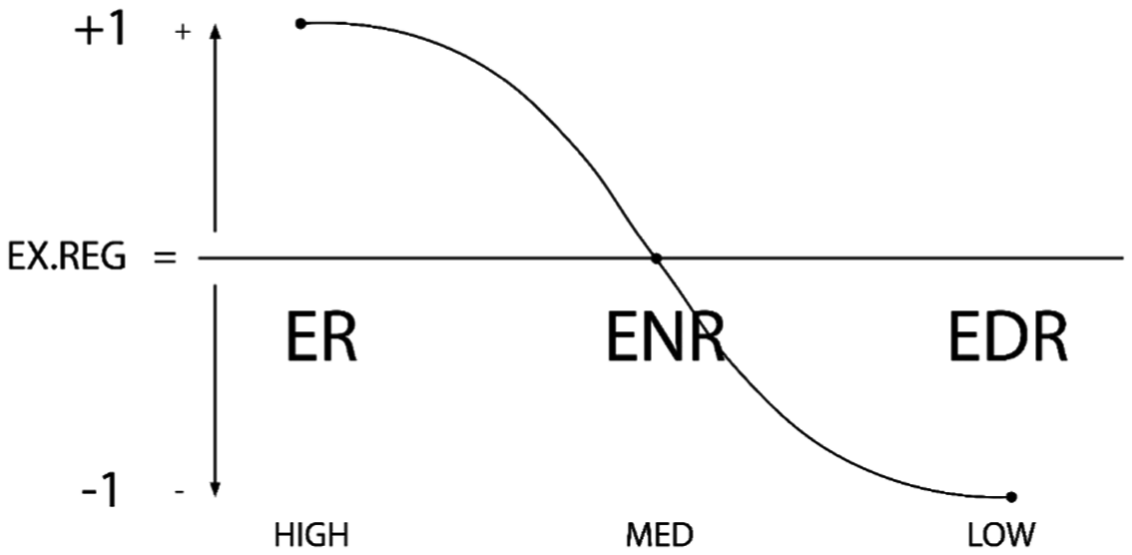
Graphic representation of the types of external regulation: ER (External Regulation), ENR (External Non-Regulation or De-regulation) and EDR (External Dys-Regulation). The degree of external regulation (high-medium-low) is plotted on the x axis and the y axis shows the positivity/negativity of external regulation (+1, 0, −1). The curved line shows the possible types of the context regulatory types and the possible directionality of the change in types of contexts.

*Principle and Hypothesis 2: Types of External Regulation*. Context factors are also considered proximal or influential when determining the variability of this behavior, with the *External-Regulation* Behavior (ER), *External Non-Regulation or De-regulation* behavior (ENR), and *External Dys-Regulation* behavior (ER) typologies:

4. *External-Regulation Behavior Type* (SR): It refers to the design and the characteristics of the context (such as antecedents and consequences of behavior), which probabilize and help exercise behavioral self-regulation (thoughts, emotions, and actions). It is considered an context adaptive and positively proactive behavioral level (ER = +1).
5. *External Non-Regulation or External De-regulation Behavior Type* (NR): It refers to the design and the characteristics of the context (such as antecedents and consequences of behavior), which do not externally probabilize or help self-regulation or dys-regulation; that is, the design of the context leaves the entire weight of regulation in the hands of the person. It is considered a context reactive or neutral contextual behavioral level in positive and negative proactivity (ER = 0).
6. *External Dys-Regulation Behavior Type* (DR): It refers to the design and the characteristics of the context (such as antecedents and consequences of behavior), which make possible and help exercise behavioral dys-regulation (in thoughts, emotions, and actions), making different kinds of behavioral excesses or deficits probable. It is considered a context adaptative and negative proactive behavioral level (ER = −1). See Figure 2.

From a behavioral perspective, if a context has a pro-regulatory value that means that it promotes self-regulation through specific behavioral mechanisms: adequate understanding of the precursors to and consequences of behavior, the degree of behavioral predictability that can be inferred from the context. Such is the susceptibility of human beings to the influence of their context that context can induce or externally promote SR, NR, and DR behaviors. Thus, context can be categorized into the same three levels of external regulation: ER (External Regulation), ENR (External Non-Regulation); and EDR (External Dys-Regulation). Here too, the absence of regulation has two levels rather than just one. ER and ENR can therefore be expected to be negatively associated, whilst ENR and EDR are positively associated such that ENR is the intermediate or prior step toward EDR.

*Principle and Hypothesis 3: Internal vs. External Behavior Combination Regulation* (combined regulation). Variability in human behavioral regulation depends on the combination of personal and contextual factors. That is, on the specific combination of the subject’s levels of personal self-regulation (high-medium-low) and the regulatory levels of the contextual regulation (high-medium-low). The heuristic used has five possible combinations of self-regulation and external regulation. This hypothesis has previously been tested and validated, with considerable consistency (de la Fuente et al., 2017, 2019b). The combination of both joint levels will be able to predict the level of this behavior, in different areas of behavior, for example, the clinical, educational, health, or technological field. The categories of high-medium-low behavioral combination of the subject and the context define 5 types of possible heuristic levels, already reported previously (de la Fuente, 2017; de la Fuente et al., 2022a). See Table 1.

**Table 1:** Combinations of the model parameters hypothesized by SRL vs. ERL Theory (de la Fuente et al., 2019a).

Combination level		Regulation average/rank		Regulation tendency	Protection level	Risk level
SR Level (range)	RT level (range)					
**3** (3.85–5.00)**H**	**3** (3.84–5.00)**H**	3.0	**5**	**High-High:** *High-Regulation*	*High protector*	*Low risk*
**2** (2.34–3.84)**M**	**3** (3.85–5.00)**H**	2.5	**4**	**Medium-High:** *Regulation*	*M-H protector*	*M-L risk*
**3** (3.85–5.00)**H**	**2** (2.35–3.84)**M**	2.5	**4**	**High-Medium:** *Regulation*	*M-H protector*	*M-L risk*
**2** (2.34–3.84)**M**	**2** (2.35–3.84)**M**	2.0	**3**	**Medium-Medium:** *Non-Regulation*	*Medium protector*	*M risk*
**2** (2.34–3.84)**M**	**1** (1.00–2.34)**L**	1.5	**2**	**Medium-Low:** *Dys-regulation*	*M-L protector*	*M- H risk*
**1** (1.00–2.34)**L**	**2** (2.35–3.84)**M**	1.5	**2**	**Low-Medium:** *Dys-regulation*	*M-L protector*	*M- H risk*
**1** (1.00–2.34)**L**	**1** (1.00–2.34)**L**	1.0	**1**	**Low-Low:** *High Dys-regulation*	*Low protector*	*High risk*

The type and level of personal and contextual regulation is calculated through cluster analysis, to delimit the low-medium and high groups. The values in parentheses mark the upper and lower cut-off points of each group. Group 1 = Low; group 2 = Medium; group 3 = High; the average of both types gives rise to a regulation average and a regulation ranking between 1 and 5.

**Figure 3:**
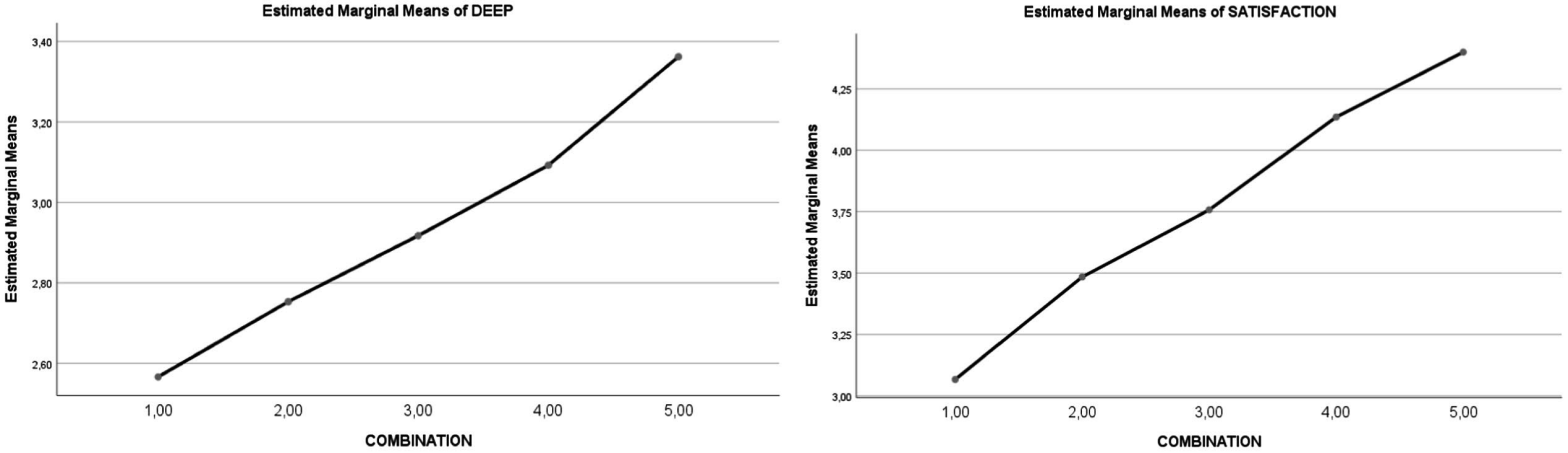
Effect of the SR-ER couple on the variables of *deep learning* and *satisfaction in learning.*

A graphical presentation of the SR-ER combination can be seen in a number of published works which have repeatedly corroborated the same trend (de la Fuente et al., 2017). See Figure 3.

### Self-regulation vs. external behavior regulation (SR-ER) in clinical psychology contexts

#### Self-regulation (SR) in clinical psychology contexts

In the field of clinical research, the *self-regulation* variable has appeared to be important for the explanation of other psychological constructs, such as personality (Inzlicht et al., 2021), resilience (de la Fuente et al., 2017), personal strengths (Lerner et al., 2021), coping strategies (Amate-Romera and de la Fuente, 2021), emotionality (Lajoie et al., 2021) and perfectionism (Thakre and Sebastian, 2021). Recently, studies have considered the dysfunctional level of self-regulation (*dys-regulation*) as a transdiagnostic variable (*p factor*) underlying numerous psychiatric psychopathologies (Duncan et al., 1996; Choi and Abbott, 2020; Huffhines et al., 2020; Smith et al., 2020; Levin-Aspenson et al., 2021; Romer et al., 2021) varying levels of which are relevant to criminal pathologies (Billen et al., 2021).

#### Internal vs. external self-regulation, non-regulation, dys-regulation (SR-ER) behavior in clinical psychology contexts

The SR-ER theoretical model (de la Fuente, 2017) proposes that the interaction of each person’s SR-NR-DR levels with their contextual ER-ENR-EDR levels is predictive and explanatory of *adaptive vs. maladaptive* behaviors for which explanation is sought. Thus, that interaction has been shown to determine the level of the variables of psychological reactance (Pachón-Basallo et al., 2021), procrastination (de la Fuente et al., 2021b), symptoms of stress and anxiety (de la Fuente et al., 2021b), positive–negative affects and psychological well-being and executive functioning and emotional dysregulation (Leerkes et al., 2020; de la Fuente et al., 2022b) with repeated consistent effects. In each case, the five-level SR-ER combinatory heuristic shows discriminatory power to determine the level of the dependent variables measured. Recent research has also shown the dysregulatory effect of traumatic experiences in childhood and adolescence, because they have produced regulatory imbalances, producing cognitive, emotional, and behavioral excesses or deficits (Claudine et al., 2021).

### Self-regulated vs. externally regulated learning (SRL–ERL) in educational psychology contexts

#### SR in educational psychology contexts

In the field of education, SR research has focused on the *Self-Regulated Learning* (SRL) construct. Historically, different theoretical models of SRL have coexisted (Panadero, 2017). Of those competing models, one of the most successful in determining the specific behavioral levels of the learning process is the model put forward by Zimmerman (Zimmerman and Schunk, 2001).

There is extensive evidence available in relation to the role of SRL in education and educational processes. SRL has been shown to be associated with numerous aspects of the learning process: motivation, emotion, and performance (de la Fuente and Eissa, 2010; Peña-Lara, 2015; Dinsmore et al., 2020). Those associations have also been shown in different stages of education, in particular university education (de la Fuente et al., 2017).

Recent studies have also shown that SR is a personality variable that suggests or predicts SRL (de la Fuente et al., 2015), academic emotions (de la Fuente et al., 2020a), coping strategies (de la Fuente et al., 2020c), and levels of academic stress (de la Fuente et al., 2020c).

#### Internally vs. externally regulated, unregulated, and dysregulated learning (SRL-ERL) behavior in educational psychology contexts

A number of studies have considered SRL-ERL, some of which have gone as far as to propose that the environment has a greater regulatory value than the subject in computer contexts (Azevedo et al., 2005). Earlier work by our research team using the SRL-ERL model (de la Fuente, 2017) also showed how the combination of the low-medium-high SR level of students and the RT level of the teaching process produces an effect with a stable linear rising function for the five levels described. That linear function determines the level of other dependent variables, such as emotions associated with academic achievement (de la Fuente et al., 2020a), learning focus (de la Fuente et al., 2020c), academic confidence (de la Fuente et al., 2021c), and protective and risk factors for stress (de la Fuente, 2021a) in a recurrent manner.

### Self-regulation vs. external regulation in psychology of health (SRH-ERH) contexts

#### SR and psychology of health contexts

Research into SR has also been significant in the field of health. SR has been integrated into models of a number of health problems and their prevention (Hull and Slone, 2004; Blood, 2012; Mann et al., 2013; Rathnayake and Chandradasa, 2020). The positive predictive value of SR in relation to health has been confirmed (Quinn and Fromme, 2010; Garzón-Umerenkova et al., 2017). Evidence in relation to the role of SR in chronic disease in the field of the Psychology of Health is extensive (Hennessy et al., 2020; Wilson et al., 2020). The SR model has also been used in specific pathologies (Clark and Zimmerman, 1990; Zimmerman et al., 1999).

Generally speaking, earlier evidence has in common the secondary or indirect value attributed to context in the explanation of the probability of different behaviors, although some recent work has considered context (Höhn et al., 2020). Hence the need to widen the focus of our vision, moving from the molecular to the molar, to pay closer attention to the interaction between the person and their environment (de la Fuente et al., 2020a).

#### Internal vs. external self-regulation, non-regulation, dys-regulation (SRH-ERH) behavior in psychology of health contexts

Earlier research has shown the harmful effects of dysregulatory contexts on psychological well-being. Other earlier studies have consistently shown that the *SR-ER combination* of the low-medium-high SR level of students and of the teaching process produces an effect with a linear function—that rises or fall depending on the variable—for the five levels described. That linear function determines in a recurrent format the level of other dependent variables such as the factors leading to, and the symptoms of, academic stress (de la Fuente et al., 2020c) and the coping strategies used (de la Fuente et al., 2020a).

### Self-regulation vs. external regulation In psychology of technology (SRT-ERT) contexts

#### SR and psychology of technology contexts

SR has appeared to be an important variable in determining how appropriately technology is used that offers a degree of protection against addictive behaviors (Chen et al., 2021; Khan et al., 2021). Alongside that, there is considerable prior research that provides systematic evidence that an individual’s level of’ behavioral self-regulation (impulsiveness and lack of control) affects and may determine where on the appropriate use-abusive dependent use of technology continuum the individual falls (Azevedo and Feyzi-Behnagh, 2011). In fact, the term “behavioral addiction” was coined to refer to the problem and the maladjustment inherent to lack of self-regulation in the use of today’s technological devices (Kuss et al., 2014; Maya, 2020).

#### Internal vs. external self-regulation, non-regulation, dys-regulation (SRH-ERH) behavior in psychology of technology contexts

Contextual factors have also been associated with or predictive of technology-related addictive behaviors (Li, 2021). However, we know relatively little about the role of the interaction of the individual with their context in terms of the fostering of maladjusted behavior in the use and abuse of technology. Knowledge of the interaction between an individual’s level of self-regulation (SR-NR-DR) and their context (ER-ENR-ED) could materially advance our knowledge of the relative contribution of combinations of those factors to explaining the variability of addictive vs. non-addictive use of technology. The different levels predicted by this new theoretical model have yet to be shown.

### Aims

Against that theoretical background, the objectives of this research were: (1) to provide empirical validation of the (internal and external) SR-NR-DR continuum proposed by using the instrument put forward; (2) to validate the different versions of the tool, as applied to different psychological contexts: clinical, educational, health and technology. The assumed *hypotheses* were: (1) The total scores for the different versions of the instrument would share a construct structure and acceptable levels of reliability in the continuum proposed and would have sufficient discriminant validity to categorize the different types of combination proposed in the SR-ER combination: 1. Low; 2. Medium-Low; 3. Medium; 4. Medium-High; 5. High. (2) The different versions of the instrument would have adequate construct validity and reliability with sufficient discriminant power or external validity with respect to different constructs of relevance in each field: clinical, educational, health, and technology.

## Materials and methods

### Participants

A total sample of 1,358 (770 women and 558 men) carry out was obtained through convenience sampling, from Spanish university students attending public universities. The students were studying different academic subjects at different levels. The age range was 18–25 (mean = 22.50; dt = 1.90). Each scale was completed by an average of 489 students. The sample was randomly divided into two subsamples (50 and 50%) in order to carry out parallel studies that would allow corroborating and verifying the results obtained (cross validation). The first half (subsample 1) was made up of 680 students: 390 women and 294 men. The second half (subsample 2) was made up of 678 students: 380 women and 264 men.

### Instruments

Self-regulation vs. external regulation behavior (de la Fuente, 2022; See Supplementary Material).

1. *Self-Regulation vs. External Regulation in Clinical Psychology Contexts (ER vs. ER)*. This variable was measured using the *Self-Regulation vs. External Regulation Scale* (de la Fuente, 2022). The scale consists of a total of 36 items self-reported against a Likert scale (1 = does not apply to me, 5 = very much applies to me). It has six components each formed of six items, through which both the behavioral types, Self-Regulation Behavior (SR), Non-regulation Behavior (NR), and Dys-Regulation Behavior (DR), and the contextual types, external regulation behavior (ER), External Non-regulation behavior (ENR), and External dys-regulation Behavior (EDR) are measured.
2. *Self-Regulated vs. Externally Regulated Learning Behavior in Educational Psychology Contexts (SRL vs. ERL).* This variable was measured using the *Self-Regulated vs. Externally Regulated Learning Scale in Educational Psychology* (de la Fuente, 2022). This scale consists of a total of 36 items self-reported against a Likert scale (1 = does not apply to me, 5 = very much applies to me). It contains six factors each formed of six items through which both the behavioral types SRL (Self-Regulated Learning), NRL (Non-Regulated Learning) and DRL (Dys-Regulated Learning), and the contextual types ERL (Externally Regulated Learning), ENRL (Externally Non-Regulated Learning) and EDRL (Externally Dys-Regulated Learning) are measured.
3. *Self-Regulation vs. External Regulation Behavior in Health Psychology Contexts (SRH vs. ERH)*. This variable was measured using the *Self-Regulation vs. External Regulation Scale in Health Psychology Contexts* (de la Fuente, 2022). This scale consists of a total of 36 items self-reported on a Likert scale (1 = does not apply to me, 5 = very much applies to me). It has six components each formed of six items through which both the behavioral types SRH (Self-Regulation in Health), NRH (Non-Regulation in Health) and DRH (Dys-Regulation in Health), and the contextual types ERH (External Regulation in Health), ENH (External Non-Regulation in Health) and EDH (External Dys-Regulation in Health) are measured.
4. *Self-Regulation vs. External Regulation Behavior in Technology Contexts*. This variable was measured using the *Self-Regulation vs. External Regulation Scale in Technology Contexts* (de la Fuente, 2022). This scale consists of a total of 36 items self-reported on a Likert scale (1 = does not apply to me, 5 = very much applies to me). It has six components each formed of six items through which both the behavioral types SRT (Self-Regulation in Technology), NRT (Non-Regulation in Technology), and DRT (Dys-Regulation in Technology), and the contextual types ERT (External Regulation in Technology), ENT (External Non-Regulation in Technology), and EDT (External Dys-Regulation in Technology) are evaluated. See Table 2.

**Table 2:** Table-summary of the types of regulation in the scales.

Type of Regulation	Self-Regulation	Non-Regulation	Dys-Regulation	External Regulation	External Non-Regulation	External Dys-Regulation
Clinical Psychology	SR	NR	DR	ER	ENR	EDR
Educational Psychology	SRL	NRL	DRL	ERL	ENRL	EDRL
Health Psychology	SRH	NRH	DRH	ERH	ENRH	EDRH
Technological Psychology	SRT	NRT	DRT	ERT	ENRT	EDRT
N° items	6	6	6	6	6	6
Level	Personal	Personal	Personal	Contextual	Contextual	Contextual

*Self-Regulation Behavior.* This variable was measured using the *Short Self-Regulation Questionnaire* (SSRQ), based on the original Self-Regulation Questionnaire. It has previously been validated in Spanish samples (Pichardo et al., 2014), and has acceptable validity and reliability values comparable to those of the English version. The SSRQ is composed of four factors (goal setting-planning, perseverance, decision-making, and learning from mistakes) and 17 items (all of them with saturations greater than 0.40), with a consistent confirmatory factor structure (Chi-square = 845,593, df = 113, CH/DF = 7.48; *p* < 0.001; RMR = 0.0299; NFI = 0.959, RFI = 0.951, IFI = 0.964, TLI = 0.957; CFI = 0.964; RMSEA =0.06). Internal consistency was acceptable for all questionnaire items collectively (*α* = 0.811) and for the factors of goal setting-planning (*α* = 0.709), perseverance (*α* = 0.735), and decision making (*α* = 0.757), and learning from mistakes (*α* = 0.703).

*Negative Emotional Reactivity. The Perth Emotional Reactivity Scale, PERS* (Becerra et al., 2017). This scale measures domains such as positive and negative emotional reactivity, it comprises 30 items and has a consistent confirmatory factor structure (Chi-square = 26.054, df = 5, CH/DF = 5.211; *p* < 0.001; RMR = 0.039; NFI = 0.954, RFI = 0.916, IFI = 0.962, TLI = 0.958; CFI = 0.961; RMSEA = 0.08). Reliability coefficients are Alpha total = 0.878, Omega = 0.846; Alpha 1 = 0.775, Alpha 2 = 0.797; Spearman–Brown = 0.867; Guttman = 0.867.

*Psychological Well-Being*. We used the *Scales of Psychological Well-Being* (Ryff, 1989) in Spanish (Díaz et al., 2006) in the 29-item version which has a consistent confirmatory factor structure (Chi-square = 845,593, df = 113, CH/DF = 7.48; *p* < 0.001; RMR = 0.029; NFI = 0.937, RFI = 0.942, IFI = 0.961, TLI = 0.956; CFI = 0.964; RMSEA = 0.05). The scale has six sub-scales: self-acceptance, positive relationships, autonomy, environmental mastery, personal growth, and purpose in life. We used a six-point Likert scale from “Does not apply to my life at all” to “Totally applicable.” Reliability coefficients are appropriate: Alpha total = 0.905, Omega = 0.886; Alpha 1 = 0.823, Alpha 2 = 0.832; Spearman–Brown = 0.867; Guttman = 0.867.

*Achievement Emotion (Studying)*. *Learning-Related Emotions* (de la Fuente et al., 2015). The psychometric properties of LRE were satisfactory in students from Spain. In this sample, the model obtained good fit indices. Unidimensionality of the scale and metric invariance were confirmed in the samples evaluated (Chi-square = 10,885.597, Degrees of freedom = 3,052, *p* < 0.001; CFI = 0.959, TLI = 0.942, IFI = 0.969, TLI = 0.955, and CFI = 0.958; RMSEA = 0.038; HOELTER = 501, *p <* 0.05; 511 *p <* 0.01). Reliability coefficients are appropriate [Cronbach Alpha = 0.930, omega = 0.897; part 1 = 0.880 (38 items), and part 2 = 0.846 (37 items), respectively, for each part (75 items)].

*TABP: Impatience-Hostility. Action-emotion style. The Jenkins Activity Survey for Students – Form H (JASE-H)* was used. This scale for measurement of TABP was adapted (Bermúdez et al., 1990, 1991) from the form T Jenkins Activity Survey (Krantz et al., 1974). It measures four components: Impatience, Hostility, Competitiveness, and Overwork. In total, the questionnaire contains 32 items, each with a six-point Likert-type response format. The subject has to choose the degree to which an item applies to them, where 1 means that the item does not apply at all to the subject and 6 means that it is fully applicable. The JASE-H offers both a global TABP score, obtained by adding the scores for all the items, and specific measurements for each component of the TABP. The JASE-H shows high internal consistency (alpha coefficient of 0.85 for the total scale, 0.81 for Impatience-Hostility, 0.82 for Competitiveness, and 0.70 for Overwork) and high stability over time, both for the complete scale (0.68) and for each subscale (0.61, 0.76 and 0.70, respectively). Reliability and validity measurements reported by the authors are consistent. The statistics are Alpha = 0.832, Omega = 8.031; and Guttman Split-Half = 0.803.

### Procedure

In five different studies, students completed their questionnaires (see Complementary Material) on an online platform: www.inetas.net (de la Fuente et al., 2015), after signing an informed consent form. Different students completed five specific questionaries during a two-year academic period. *Inventory 1* was assessed in September–October of 2019 and 2020; *Inventory 2*, in November–December of 2019 and 2020; *Inventory 3*, in February–March of 2019 and 2020; *Inventory 4*, in April–May of 2019 and 2020; and *Inventory 5* variables in May–June of 2019 and 2020. The *Self-Regulation Questionnaire* was completed with the other questionnaires in April–May 2019–2020. Questionnaire completion was voluntary. The respective Ethics Committees of the two universities approved the procedure as part of an R&D Project (2018–2021): http://www.estres.investigacion-psicopedagogica.org/lib/pdf/CERTIFICADO_COMITE_DE_ETICA_UNAV.pdf.

### Data analysis

*Sample design.* A random sample was designed to estimate the proportion of interest if measured at a level that is greater than 200 people (*n* > 200); that is, the maximum permissible error in the estimation of the proportions of 7% and equivalently for the estimation of the average score of the scale.

*Content validity: through expert validity.* The methodological reference for the process of content validity by expert judgment was considered as “an informed opinion of people with experience in the subject, who are recognized by others as qualified experts in it, and who can provide information, evidence, judgments and assessments” (Escobar and Cuervo, 2008; p. 29). A template was used, developed by these authors, with four categories, and a licker-type response range from 1 (not at all) to 5 (a lot):

1. *Clarity*: the items are understood correctly, with adequate syntax and semantics.
2. *Coherence:* the items have an adequate relationship with the dimension and scale.
3. *Relevance:* the items are completely related to the dimension and scale under analysis.
4. *Sufficiency:* the items of each dimension are sufficient to measure it adequately.

This template was sent to seven experts on the topic (self-regulation), from each area and type of Scale. They were considered so if they were accredited by their research experience with more than 10 recently published articles on the topic. Upon receipt, a content validity coefficient analysis was applied by degree of interjudge agreement per item. A degree of agreement of 80% was obtained in the items of each scale, which was considered acceptable, es decir un IFV de 0.80 (Rubio et al., 2003).

*Preliminary analysis*. Adequacy of parametric analyses was first confirmed by determination of normal distribution (Kolmogorov–Smirnov test), skewness, and kurtosis (+/−0.05). In this case, the majority of values were below or near 0.50.

*Criterion or concurrent validity: Correlation*. For purposes of evaluation of the associations posited by the study hypotheses, positivity was correlated with resilience, coping strategies, and engagement-burnout (Pearson bivariate correlation) using SPSS (v.26). The assumptions for the bivariate correlation were: (1) The data have a linear relationship as established by scatter plot; (2) The variables are normally distributed; (3) The observations used for the bivariate correlation are a random sample from the reference population. Correlation bands were set according to customary criteria: low (0.10–0.30), medium (0.40–0.70), and high (0.80–0.90).

*Construct validity*. The sample was randomly divided into two subsamples (50 and 50%) using the Statistical Package for the Social Sciences (SPSS, version 26) in order to carry out parallel studies that would allow corroborating and verifying the results obtained (cross validation):

1. *Exploratory Factorial Analysis (EFA).* This analysis was performed with 50% of the sample. The Kaiser–Meyer–Olkin indices, Bartlett’s Sphericity Test, and factor communality values were used. Varimax rotation was used, with maximum likelihood and percentage of variance explained by each factor and the total of the scale. KMO was taken to be 0.80 and the Bartlett significance level was *p* < 0.001.
2. *Confirmatory Factorial Analysis (CFA)*. With the remaining 50% of the sample, the previous factorial structure was calculated. Model fit was assessed by the Chi-square: degrees of freedom ratio, Comparative Fit Index (CFI), Normed Fit Index (NFI), Incremental Fit Index (IFI), and Relative Fit Index (RFI). Target values were greater than 0.90. We used the Hoelter Index to confirm that the sample was of adequate size (Tabachnick and Fidell, 2001). AMOS (v.26) was used.

*Reliability.* Cronbach’s Alpha index and the Omega Index (McDonald, 1999) were used. Cut-off values were set at 0.80.

*Variance Analysis*. ANOVA and MANOVA were performed to analyze external and concurrent validity. First, each subject’s score for regulation in each questionnaire was calculated as: *Total Internal and External Regulation* = [(SR + ER)/2 − (SNR + ENR)/2 − (SDR + EDR)/2)]/3. This continuous heuristic is adjusted to a linear format (see Figures 1, 2) with respect to the previous scalar heuristic (see Table 1). Subsequent cluster analysis determined the central values and the intersection points between them for each questionnaire and for the questionnaires as a whole. As can be seen, the distribution of the inventories follows the curve of the proposed theoretical relationship, albeit in a wider range of approximately −2.00–1.00. This comes about because levels of regulation are totaled; thus, whilst self-regulation is positive (+1.00), non-regulation and dys-regulation are negative (up to −2.00 at most). It should be noted that the scores in the table are similar for the different scales and the General Scale. See Table 3.

**Table 3:** Central and limit value for the clusters for each questionnaire and general.

Context	Inventory	Limit	Level 5	Limit	Level 4	Limit	Level 3	Limit	Level 2	Limit	Level 1	Limit
Clinical Psychology	SR-ER	1,00	**0.64**	0.39	**0.15**	0.08	**−0.29**	−0.49	**−0.70**	−0.92	**−1.15**	−2.00
Education Psychology	SRL-ERL	1,00	**0.68**	0.44	**0.21**	−0.08	**−0.37**	−0.63	**−0.92**	−1.11	**−1.33**	−2.00
Health Psychology	SRH-ERH	1,00	**0.72**	0.51	**0.30**	0.05	**−0.20**	−0.48	**−0.77**	−1.00	**−1.23**	−2.00
Technology Psychology.	SRT-ERT	1,00	**0.57**	0.33	**0.10**	−0.13	**−0.37**	−0.60	**−0.84**	−1.06	**−1.28**	−2.00
General	SRG-ERG	1,00	**0.62**	0.38	**0.14**	−0.10	**−0.35**	−0.59	**−0.84**	−1.06	**−1.28**	−2.00
(*n* = 2,716)			(*n* = 358)		(*n* = 516)		(*n* = 750)		*n* = 742		*n* = 350	

Level 5, High; Level 4, Medium-high; Level 3, Medium; Level 2, Medium-low; Level 1, Low. The levels (5,4,3,2,1) in each inventor are highlighted in bold.

## Results

### Study 1. Self-regulation vs. external regulation behavior psychology total inventory (SRT-ERT)

#### Descriptive results

The descriptive values found met the normality requirements to be expected of this type of sample and subsequent analysis. See Table 4.

**Table 4:** Values descriptive of the total validation sample (*n* = 1,358).

Variable	Range	Mean (dt)	Deviation error	Asymmetry	Dev. Error	Kurtosis	Dev. Error
SRTOT	1–5	3.96 (0.739)	0.018	−0.592	0.060	0.209	0.119
NRTOT	1–5	2.67 (0.760)	0.018	0.245	0.060	−0.072	0.119
DRTOT	1–5	2.49 (0.911)	0.022	0.295	0.059	−0.441	0.119
ERTOT	1–5	3.76 (0.934)	0.023	−0.530	0.061	−0.230	0.121
ENRTOT	1–5	2.51 (0.957)	0.023	0.234	0.060	−0.577	0.121
EDRTOT	1–5	2.42 (1.02)	0.025	0.323	0.060	−0.710	0.121

SRTOT, Self-Regulation Behavior Total; NRTOT, Non-Regulation Behavior Total; DRTOT, Dys-Regulation Behavior Total; ERTOT, External Regulation Behavior Total; ENRTOT, External Non-Regulation Behavior Total; EDTOT, External Dys-Regulation Behavior Total.

#### Construct validity

##### Correlation

SRTOT was negatively correlated with NRTOT and DRTOT; NRTOT and DRTOT had a significant negative correlation. Across this context, the correlations are the same in terms of direction: negative between ERTOT and ENTOT and positive between ENTOT and EDTOT. Note also the consistent negative and positive correlation of components of the scale with the aggregate score for the SR-ER.TOT construct. See Table 5.

**Table 5:** Correlation between internal and external regulation and the total score for the scale (*n* = 1,358).

	SRTOT	NRTOT	DRTOT	ERTOT	ENTOT	EDTOT
SRTOT						
NRTOT	−0.220**					
DRTOT	−0.053*	0.648**				
ERTOT	0.500**	−0.113**	−0.015			
ENTOT	−0.175**	0.617**	0.582**	−0.265**		
EDTOT	−0.033	0.572**	0.703**	−0.021	0.681**	
SR-ER.TOT	0.463**	−0.770**	−0.749**	0.478**	−0.843**	−0.763**

SRTOT, Self-Regulation Behavior Total; NRH, Non-Regulation Behavior Total; DRH, Dys-Regulation Behavior Total; ERTOT, External Regulation Behavior Total; ENRTOT, External Non-Regulation Behavior Total; EDRTOT, External Dys-Regulation Behavior Total; **p < 0.001; *p < 0.01.

*Exploratory Factorial Analysis* (EFA). This analysis was carried out with 50% of the sample, obtaining adjusted values: Kaiser–Meyer–Olkin = 0.936; Bartlett’s Sphericity Test (630) = 15,703, 146, *p <* 0.001; factor communality was between 0.426 (item 8) and 0.785 (item 34). In the varimax rotation, six factors appeared that explained 65% of the variance: Factor 1, *EDRT* (24.5% variance) = items 34, 33, 35, 36, 32, 31; Factor 2, *ERT* (13.14% variance) = 21,23,20, 24, 22, 19; Factor 3, *SRT* (14.05%) = 3,4,5,6,2,1; Factor 4, *DRT* (11.54% variance) = 16,15,13, 17,18, 14; Factor 5, *NRT* (10.64%) = 29, 28, 27,25,26,30; Factor 6, *NRT* (5.71%) = 10,7,9,8,11,12.

*Confirmatory Factorial Structure.* The structural values for this construct appeared to be acceptable [Chi-square = 3,527.914, *p* < 0.001; df (702–118) = 584; CH/DF = 6.041; CFI = 0.912; GFI = 0.900; IFI = 0.926; TLI = 0.915; CFI = 0.926, RMSEA = 0.019; RSMR = 0.045; Hoelter = 2,417 (*p* < 0.05), 2,512 (*p* < 0.01)]. See Table 6.

**Table 6:** Standardized total effects (default model; *n* = 840).

	F1	F2	F3	F4	F5	F6
SRERTOT1	0.696					
SRERTOT2	0.754					
SRERTOT3	0.824					
SRERTOT4	0.818					
SRERTOT5	0.789					
SRERTOT6	0.609					
SRERTOT7		0.591				
SRERTOT8		0.331				
SRERTOT9		0.704				
SRERTOT10		0.677				
SRERTOT11		0.777				
SRERTOT12		0.528				
SRERTOT13			0.651			
SRERTOT14			0.625			
SRERTOT15			0.699			
SRERTOT16			0.771			
SRERTOT17			0.771			
SRERTOT18			0.735			
SRERTOT19				0.790		
SRERTOT20				0.860		
SRERTOT21				0.887		
SRERTOT22				0.880		
SRERTOT23				0.894		
SRERTOT24				0.845		
SRERTOT25					0.632	
SRERTOT26					0.653	
SRERTOT27					0.789	
SRERTOT28					0.849	
SRERTOT29					0.828	
SRERTOT30					0.705	
SRERTOT31						0.778
SRERTOT32						0.795
SRERTOT33						0.811
SRERTOT34						0.844
SRERTOT35						0.808
SRERTOT36						0.818

#### Criterion-related validity: SR-ER general

*Formation of groups.* The ANOVA carried out to form the groups showed a significant principal Group Factor effect for SR-ER TOTAL relative to the total score for SR-ER.TOT [*F*(4.1353) = 5430.739, *p* < 0.001; *eta*^2^ = 0.941, *power* = 1.00; *post-hoc* = 5 > 4 > 3 > 2 > 1, *p* < 0.001]. Levene’s test of error variance based on the mean showed the adequacy of the groups [*L*(4.1353) = 1.949, *p* < 0.127]. See Table 7 for the descriptive statistics.

**Table 7:** Standardized total effects (default model; *n* = 360).

	F1	F2	F3	F4	F5	F6
SRERL1	0.670					
SRERL2	0.786					
SRERL3	0.807					
SRERL4	0.873					
SRERL5	0.820					
SRERL6	0.623					
SRERL7		0.511				
SRERL8		0.313				
SRERL9		0.730				
SRERL10		0.702				
SRERL11		0.783				
SRERL12		0.521				
SRERL13			0.680			
SRERL14			0.662			
SRERL15			0.795			
SRERL16			0.773			
SRERL17			0.823			
SRERL18			0.728			
SRERL19				0.793		
SRERL20				0.870		
SRERL21				0.897		
SRERL22				0.871		
SRERL23				0.872		
SRERL24				0.825		
SRERL25					0.628	
SRERL26					0.632	
SRERL27					0.750	
SRERL28					0.822	
SRERL29					0.833	
SRERL30					0.724	
SRERL31						0.819
SRERL32						0.817
SRERL33						0.850
SRERL34						0.849
SRERL35						0.784
SRERL36						0.796

*Effect of the SR-ER General group on each type of regulation*. The ANOVA carried out showed a significant principal effect of the SR-ER General Group relative to each FACTOR IN TOTAL REGULATION [*F*(24.5404) = 77.493 (Pillai), *p* < 0.001; *eta*^2^ = 0.256, power = 1.00], and to the individual components: *SRT* [*F*(4,1,353) = 93.301, *p* < 0.001; *eta*^2^ = 0.216, power = 1.00]; *NRG* [*F*(4.1353) = 93.301, *p* < 0.001; *eta*^2^ = 0.561, power = 1.00]; *DRT* [*F*(4.1353) = 387.232, *p* < 0.001; *eta*^2^ = 0.534, power = 1.00]; *ERT* [*F*(4,1,353) = 93.301, *p* < 0.001; *eta*^2^ = 0.261, power = 1.00]; *ENRT* [*F*(4.1353) = 93.301, *p* < 0.001; *eta*^2^ = 0.676, power = 1.00]; *EDRT* [*F*(4.1353) = 93.301, *p* < 0.001; *eta*^2^ = 0.556, power = 1.00]. Note the greater explanatory weight of the indices in both the internal and external non-regulation and dys-regulation components. Levene’s test of error variance based on the mean showed the adequacy of the groups [*L*(4.1353) = 2.788, *p* < 0.099]. See Table 8 for the descriptive statistics.

**Table 8:** Descriptive statistics for the groups formed with the total scores (*n* = 1,385).

SR-ER TOT groups	*n* = 1,385	Mean	SRTOT	NRTOT	DRTOT	ERTOT	ENRTOT	EDRTOT
1. LTOT	185	−1.26 (0.15)	3.63 (0.85)	3.67 (0.58)	3.61 (0.73)	3.27 (0.81)	3.79 (0.57)	3.61 (0.82)
2. MLTOT	365	−0.836 (0.13)	3.70 (0.72)	3.01 (0.48)	2.92 (0.60)	3.46 (0.96)	3.01 (0.54)	2.93 (0.70)
3. MTOT	374	−0.345 (0.14)	3.94 (0.63)	2.57 (0.50)	2.35 (0.65)	3.61 (0.89)	2.39 (0.60)	2.30 (0.74)
4. MHTOT	258	−0.137 (0.13)	4.26 (0.58)	2.20 (0.48)	1.93 (0.59)	4.14 (0.77)	1.76 (0.50)	1.68 (0.61)
5. HIGTOT	179	618 (0.17)	4.60 (0.43)	1.77 (0.45)	1.41 (0.43)	4.72 (0.44)	1.25 (0.35)	1.17 (0.28)
*Mean*			3.95 (0.78)	2.66 (0.73)	2.47 (0.90)	3.75 (0.94)	2.48 (0.28)	2.38 (0.1.01)
*Post-hoc*			5 > 4 > 3 > 2.1^**^	5 < 4 < 3 < 2 < 1^**^	5 < 4 < 3 < 2 < 1^**^	5 > 4 > 3 > 2.1^**^	5 < 4 < 3 < 2 < 1^**^	5 < 4 < 3 < 2 < 1^**^

LTOT, Low; MLTOT, Medium-Low; MTOT, Medium; MHTOT, Medium-High; HIGTOT, High; SRTOT, Self-Regulation Total; NRTOT, Non-Regulation Total; DRTOT, Dys-Regulation Total; ERTOT, External-Regulation Total; ENRTOT, External Non-Regulation Total; EDRTOT, External Dys-Regulation Total; ^**^p < 0.001.

#### Reliability

The total reliability of the scale showed adequate ratios (Cronbach’s Alpha = 0.900; Omega Index = 0.897). Split-half analysis showed adequate values (Alpha 1 = 0.802; Alpha 2 = 0.858; Spearman–Brown Coefficient = 0.828; Guttman Split-half Coefficient = 0.828). The ratios for each scale also: SR (Alpha = 0.888), NR (Alpha = 0.738), DR (Alpha = 0.857), ER (Alpha = 0.943) ENR (Alpha = 0.880); EDR (Alpha = 0.918).

### Study 2. Self-regulation vs. regulatory behavior inventory regulation in *clinical psychology* contexts (SR-ER)

#### Descriptive results

The descriptive values found met the normality requirements to be expected of this type of sample and subsequent analysis. See Table 9.

**Table 9:** Values descriptive of the validation sample (*n* = 422).

Variable	Range	Mean (dt)	Deviation error	Asymmetry	Dev. Error	Kurtosis	Dev. Error
SR	1–5	4.00 (0.686)	0.033	−0.579	0.119	0.765	0.237
NR	1–5	2.71 (0.723)	0.035	0.281	0.119	0.365	0.237
DR	1–5	2.62 (0.845)	0.041	0.232	0.119	−0.236	0.237
ER	1–5	4.25 (0.906)	0.045	−0.585	0.121	0.025	0.242
ENR	1–5	2.52 (0.963)	0.047	0.304	0.121	−0.562	0.242
EDR	1–5	2.55 (1.01)	0.049	0.197	0.120	−0.850	0.238

SR, Self-Regulation; NR, Non-Regulation; DRL, Dys-Regulation; ERB, External Regulation; ENR, External Non-Regulation; EDR, External Dys-Regulation.

#### Construct validity

##### Correlation

There was a significant negative correlation of SR with NR and DR and a significant positive correlation of NR with DR. Across this context, the correlations are consistent in direction: negative between ER and EN and positive between ENR and EDR. Finally, the trend seen with general *Self-Regulation* was confirmed. The correlations between the components of the scale and the scores for the total construct have the same directions. See Table 10.

**Table 10:** Correlations between the Self-Regulation General (SRG) construct and the types of self-regulation and external regulation (SR-ER; *n* = 422).

	SR	NR	DR	ER	ENR	EDR
SR						
NR	−0.111^**^					
DR	−0.086^*^	0.611^**^				
ER	0.460^**^	0.096^*^	0.151^**^			
ENR	−0.130^**^	0.590^**^	0.546^**^	−0.047		
EDR	−0.059	0.504^**^	0.643^**^	0.156^*^	0.619^**^	
SRG	0.413^**^	−0.221^**^	−0.131^**^	0.211^**^	−0.186^**^	−0.020
SR-ER	0.315^**^	−0.677^**^	−0.745^**^	0.231^**^	−0.695^**^	−0.841^**^

SR, Self-Regulated Behavior; NRL, Non-Regulated Behavior; DRL, Dys-Regulated Behavior; ER, External Regulation Behavior; ENR, External Non-Regulation Behavior; EDR, External Dys-regulation Behavior; SRG, General Self-Regulation. SR-ER, Total Self-Regulation vs. External Regulation; ^**^p < 0.001; ^*^p < 0.01.

*Exploratory Factorial Analysis* (EFA). This analysis was carried out with 50% of the sample, obtaining adjusted values: Kaiser–Meyer–Olkin = 0.876; Bartlett’s Sphericity Test (630) = 4, 154, 307, *p* < 0.001; factor communality was between 0.362 (item 8) and 0.827 (item 22). In the varimax rotation, six factors appeared that explained 65.50% of the variance: Factor 1, *EDR* (15.38% variance) = items 34, 36, 35, 32, 31, 33; Factor 2, *ER* (13.30% variance) = 23, 22, 21, 19, 24, 20; Factor 3, *SR* (11.21%) = 5, 3, 4, 6, 1, 2; Factor 4, *ENR* (10.17% variance) = 28, 29, 26, 30, 27, 25; Factor 5, *NR* (7, 78% variance) = 10, 7, 9, 11, 8, 12; Factor 6, *DR* (7.63%) = 14, 16, 17, 15, 13, 18.

*Confirmatory Factorial Analysis (CFA).* The structural values for this construct appeared to be adequate [Chi-square = 1,575.861, df (702–118) = 584, *p* < 0.001; Chi/df = 2.689; RMR = 0.0351; NFI = 0.910, RFI = 917; IFI: 938; TLI = 0.903; CFI = 0.928; RMSEA = 0.0231; HOELTER = 1,353 (*p* < 0.05) and 406 (*p* < 0.01)], showing six factors with six items each: SRL, NRL, SDL, ERL, ENRL, EDRL, with acceptable standardized effects, factorial weights adjusted. See Table 11.

**Table 11:** Standardized total effects (default model; *n* = 399).

	F1	F2	F3	F4	F5	F6
SRER1	0.646					
SRER2	0.646					
SRER3	0.813					
SRER4	0.810					
SRER5	0.801					
SRER6	0.602					
SRER7		0.607				
SRER8		0.389				
SRER9		0.690				
SRER10		0.674				
SRER11		0.747				
SRER12		0.522				
SRER13			0.507			
SRER14			0.664			
SRER15			0.501			
SRER16			0.727			
SRER17			0.772			
SRER18			0.735			
SRER19				0.794		
SRER20				0.820		
SRER21				0.895		
SRER22				0.885		
SRER23				0.902		
SRER24				0.875		
SRER25					0.596	
SRER26					0.683	
SRER27					0.815	
SRER28					0.848	
SRER29					0.841	
SRER30					0.678	
SRER31						0.741
SRER32						0.749
SRER33						0.729
SRER34						0.864
SRER35						0.804
SRER36						0.801

### Reliability

The reliability of the total Scale showed adequate ratios (Cronbach Alpha = 0.902; Omega Index = 0.896). Split-half analysis (Alpha 1 = 0.805; Spearman–Brown Coefficient = 0.828; Guttman Split-half Coefficient = 0.828). Additionally, the values for the subscales were consistent: SR (Alpha = 0.864; Omega = 0.843); SNR (Alpha = 0.717; Omega = 0.701); SDR (Alpha = 0.818; Omega = 0.802); ER (Alpha = 0.845; Omega = 0.846); ENR (Alpha = 0.877; Omega = 0.853); EDR (Alpha = 0.900; Omega = 0.878).

### External validity: Negative emotional reactivity

*Formation of groups.* The ANOVA carried out to form the groups showed a significant principal Group Factor effect for SR-ER relative to the total score for SR-ER [*F*(4.335) = 1185.439, *p* < 0.001; *eta*^2^ = 0.930, *power* = 1.00; *post-hoc* = 5 > 4 > 3 > 2 > 1, *p* < 0.001]. Levene’s test of error variance based on the mean showed the adequacy of the groups [*L*(4.355) = 2.430, *p* < 0.100]. See Table 6 for the descriptive statistics.

*Effect of the SR-ER group on the level of Negative Emotional Reactivity.* The ANOVA carried out showed a significant principal Group Factor effect for SR-ER relative to reactance [*F*(4.307) = 6.887, *p* < 0.001; *eta*^2^ = 0.08, *power* = 0.999; *post-hoc* = 5.4 > 4 > 3.2 > 2 > 1, *p* < 0.001]. Levene’s test of error variance based on the mean showed the adequacy of the groups [*L*(4.307) = 1.099, *p* < 0.357]. See Table 12 for the descriptive statistics.

**Table 12:** Descriptive statistics for the groups formed (*n* = 360).

SR-ER groups	*n* = 360	Group mean	(dt)	Lower limit	Upper limit	Negative emotional reactivity	(dt)
1. LOW	59	−1.1455	(0.133)	−0.92	−1.50	3.3224	(0.961)
2. MLOW	94	−0.7027	(0.133)	−0.49	−0.91	2.9694	(0.808)
3. MEAN	129	−0.2289	(0.166)	0.08	−0.48	2.8167	(0.973)
4. MHIGH	46	0.2403	(0.085)	0.39	0.07	2.5683	(0.964)
5. HIGH	32	0.6450	(0.161)	1.03	0.38	2.3214	(0.847)

LOW, Low; MLOW, Medium-Low; MEAN, Medium; MHIGH, Medium-High; HIGTOT, High.

### Study 3. Self-regulatory vs. external regulatory learning behavior inventory in *educational psychology* contexts (SRL-ERL)

#### Descriptive results

The descriptive values found met the normality requirements to be expected of this type of sample. See Table 13.

**Table 13:** Values descriptive for the validation sample (*n* = 360).

Variable	Range	Mean (dt)	Deviation error	Asymmetry		Dev. error kurtosis	Dev. error
SRL	1–5	3.96 (0.727)	0.032	−0.442	0.110	−0.295	0.220
NRL	1–5	2.64 (0.755)	0.033	0.260	0.110	−0.167	0.219
DRL	1–5	2.38 (0.914)	0.041	0.428	0.110	−0.374	0.219
ERL	1–5	3.71 (0.940)	0.043	−0.438	0.112	−444	0.223
ENRL	1–5	2.48 (0.940)	0.043	0.214	0.112	−0.517	0.224
EDRL	1–5	2.33 (0.990)	0.045	0.391	0.112	−0.588	0.223

SRL, Self-Regulation of Learning Behavior; NRL, Non-Regulation of Learning Behavior; DRL, Dys-Regulation of Learning Behavior.

#### Construct validity

##### Correlation

There was a significant negative correlation of SRL with NRL and DRL and a significant positive correlation of NRL and DRL. Across this context, the correlations are all in the same directions: negative for ERL with ENL and positive for ENL with EDL. Note also the positive and negative correlations between the components of SRL-ERL and the general SR construct. See Table 14.

**Table 14:** Correlation between the types of internal and external regulation and the total score for the scale (*n* = 320).

	SRL	NRL	DRL	ERL	ENL	EDL
SRL						
NRL	−0.279^**^					
DRL	−0.199^**^	0.730^**^				
ERL	0.486^**^	−0.188^**^	−0.097^*^			
ENL	−0.266^**^	0.626^**^	0.615^**^	−0.325^*^		
EDL	−0.186^**^	0.575^**^	0.706^**^	−0.122^**^	0.693^**^	
SR	0.434^**^	−0.247^**^	−0.231^**^	0.296^**^	−0.159^**^	−0.094
SRL-ERL	0.572^**^	−0.797^**^	−0.775^**^	0.551^**^	−0.846^**^	−0.769^**^

SRL, Self-Regulation in Learning; NRL, Non-Regulation in Learning; DRL, Dys-Regulation in Learning; ERL, External Regulation in Learning; ENRL, External Non-Regulation in Learning; EDL, External Dys-regulation in Learning; SR, Self-Regulation; SRL-ERL, Total Self-Regulation vs. External Regulation in Learning; ^**^p < 0.001; ^*^p < 0.01.

*Exploratory Factorial Analysis* (EFA). This analysis was carried out with 50% of the sample, obtaining adjusted values: Kaiser–Meyer–Olkin = 0.888; Bartlett’s Sphericity Test (630) = 4,782,893 *p* < 0.001; factor communality was between 0.546 (item 6) and 0.831 (item 21). In the varimax rotation, six factors appeared that explained 67.00% of the variance: Factor 1, *EDRL* (15.92% variance) = items 34, 32, 33, 35, 31, 36; Factor 2, *DRL* (13.81% variance) = 15,13,16, 14, 17, 18; Factor 3, *ERL* (13.54%) = 20, 21, 24, 23, 22 19; Factor 4, *SRL* (12.27% variance) = 4,5,2, 3, 1, 6; Factor 5, *ENRL* (7, 41% variance) = 30, 29,28, 25, 26, 27; Factor 6, *SNRL* (4.19%) = 8,10,7, 9,11,12.

### Confirmatory factor analysis

The structural values for this construct are acceptable [Chi-square = 1,598.384, df = (702–118) 584; Chi/df = 2,737; RMR = 0.0321; NFI = 0.967, RFI = 958; IFI: 918; TLI = 0.906; CFI = 0.917; RMSEA = 0.023; 1,334; 1,386], showing six components each containing six items (SRL, NRL, SDL, ERL, ENRL, EDRL), with consistent weights. See Table 7.

### Reliability

The reliability of the total Scale showed adequate ratios (Cronbach Alpha = 0.881; Omega Index = 0.876). Split-half analysis showed adequate values (Alpha 1 = 0.781; Alpha 2 = 0.831; Spearman–Brown Coefficient = 0.787; Guttman Split-half Coefficient = 0.780). The reliability of the subscales also appeared to be acceptable: SRL (Alpha = 0.897; Omega = 0.886); SNL (Alpha = 0.753; Omega = 0.732); SDL (Alpha = 0.880; Omega = 0.821); ERL (Alpha = 0.940; Omega = 0.902); ENL (Alpha = 0.877; Omega = 0.851); EDL (Alpha = 0.922; Omega = 0.901).

#### External validity: Study achievement emotions

*Formation of groups.* The ANOVA carried out to form the groups showed a significant principal Group Factor effect for SRL-ERL relative to the total score for SRL-ERL [*F*(4.385) = 1,798.369, *p* < 0.001; *eta*^2^ = 0.949, *power* = 1.00; *post-hoc* = 5 > 4 > 3 > 2 > 1, *p* < 0.001]. Levene’s test of error variance based on the mean showed the adequacy of the groups [*L*(4.385) = 1.825, *p* < 0.100]. See Table 11 for the descriptive statistics.

*Effect of the SRL-ERL group on the type and level of achievement emotion (during the study).* The ANOVA carried out showed a significant principal effect of the SR-ER Group relative to academic achievement emotions during the study [*F*(32.1072) = 4.538, *p* < 0.001; *eta^2^* = 0.119, power = 1.00]. Levene’s test of error variance based on the mean showed the adequacy of the groups [*L*(4.385) = 1.825, *p* < 0.157]. See Table 11 for the descriptive statistics. See Table 15.

**Table 15:** Descriptive statistics for the groups formed (*n* = 360).

RL-ERL groups	*n* = 360	Mean	(dt)	Enjoyment	Conf	Pride	Anger	Anxiety	Shame	Desp	Boredom
1. LOW	28	−1.31	(0.130)	3.32	3.25	3.51	3.09^*^	3.18^*^	3.02^*^	3.02^*^	3.33^*^
2. MLW	74	−0.89	(0.132)	3.29	3.30	3.49	2.44	2.80	2.34	2.34	2.62
3. EAN	74	−0.35	(0.146)	3.50	3.52	3.89	2.08	2.76	2.05	2.05	2.28
4. MH	56	0.18	(0.134)	3.70	4.03	4.14	1.62	2.19	1.47	1.47	1.79
5. HIGH	45	0.65	(0.167)	3.82^**^	4.15^**^	4.30^**^	1.41	2.15^*^	1.40	1.40	1.69

LOW, Low; MLOW, Medium-Low; MEAN, Medium; MHIGH, Medium-High; HIGTOT, High. ^**^5,4 > 3 > 2,1 positive emotions (enjoyment, confidence, pride); ^*^1,2 > 3 > 2,1 negative emotions (anger, anxiety, shame, despair, boredom).

### Study 4. Self-regulatory vs. external regulatory behavior inventory in *health psychology* context (SRH-ERH)

#### Descriptive results

The descriptive values found met the normality requirements to be expected of this type of sample. See Table 16.

**Table 16:** Values descriptive of the validation sample (*n* = 400).

Variable	Range	Mean (dt)	Deviation error	Asymmetry		Dev. error kurtosis	Dev. error
SRH	1–5	3.93 (0.782)	0.039	−0.595	0.124	0.135	0.248
NRH	1–5	2.52 (0.807)	0.040	0.378	0.123	−0.226	0.246
DRH	1–5	2.30 (0.914)	0.045	0.440	0.123	−0.368	0.245
ERH	1–5	3.81 (0.955)	0.049	−0.495	0.125	−0.343	0.250
ENRH	1–5	2.37 (0.977)	0.049	0.279	0.124	−0.790	0.247
EDRH	1–5	2.27 (1.05)	0.053	0.534	0.125	−0.505	0.249

SRH, Self-Regulation in Health; NRH, Non-Regulation in Health; DRH, Dys-Regulation in Health; ERH, External Regulation in Health; ENRH, External Non-Regulation in Health; EDRL, External Dys-Regulation in Health; **p < 0.001; *p < 0.01.

#### Construct validity

##### Correlations

There was significant negative correlation of SRH with NRH and DRH and significant positive correlation of NRH and DRH. Across this context, the correlations are consistent in direction: negative for ERH with ENH and positive for ENH with EDH. Note also the consistent negative and positive correlation of components of the scale with the SR and SR- ER constructs. See Table 17.

**Table 17:** Correlation between the types of internal and external regulation Health Psychology (*n* = 400).

	SRH	NRH	DRH	ERH	ENH	EDH
SRH						
NRH	−0.543^**^					
DRH		0.520^**^				
ERH	0.622^**^	−0.518^**^				
ENH		0.510^**^	0.516^**^	−0.509^*^		
EDH			0.617^**^		0.551^**^	
SR	0.338^**^	−0.255^**^	−0.250^**^	0.265^**^	−0.192^**^	−0.157^**^
SRH-ERH	0.513^**^	−0.785^**^	−0.792^**^	0.558^**^	−0.868^**^	−0.824^**^

SRH, Self-Regulation in Health; NRH, Non-Regulation in Health; DRH, Dys-Regulation in Health; ^**^p < 0.001; *p < 0.01.

*Exploratory Factorial Analysis* (EFA). This analysis was carried out with 50% of the sample, obtaining adjusted values: Kaiser–Meyer–Olkin = 0.892; Bartlett’s Sphericity Test (630) = 4, 459, 189 *p* < 0.001; factor communality was between 0.513 (item 6) and 0.842 (item 23). In the varimax rotation, six factors appeared that explained 70.04% of the variance: Factor 1, *EDRH* (21.60% variance) = items 34, 36, 35, 33, 31, and 30; Factor 2, *ERH* (14.97% variance) = 23, 20, 21, 22, 24, 19; Factor 3, *SRH* (11,24%) = 1, 3, 2, 4, 5, 6; Factor 4, *DRH* (10,81% variance) = 16, 15, 13, 17, 14, 18; Factor 5, *NRH* (7, 56% variance) = 9, 11, 7, 10, 12, 8; Factor 6, *ENRH* (4,19%) = 28, 26, 29, 25, 30, 31.

*Factorial Confirmatory Structure.* The structural values for this construction appeared to be acceptable [Chi-square = 1647.619, *p* < 0.001; df(702–118) = 584; CH/DF = 2,821; CFI = 0.958; GFI = 0.938; IFI = 0.926; TLI = 0.928; CFI = 0.926, RMSEA = 0.023; RSMR = 0.052; Hoelter = 1,294 (*p* < 0.05), 1,345 (*p* < 0.01)]. See Table 18.

**Table 18:** Standardized total effects (default model; *n* = 383).

	F1	F2	F3	F4	F5	F6
SRERH1	0.762					
SRERH2	0.805					
SRERH3	0.846					
SRERH4	0.811					
SRERH5	0.834					
SRERH6	0.663					
SRERH7		0.588				
SRERH8		0.378				
SRERH9		0.732				
SRERH10		0.718				
SRERH11		0.832				
SRERH12		0.594				
SRERH13			0.735			
SRERH14			0.649			
SRERH15			0.730			
SRERH16			0.834			
SRERH17			0.707			
SRERH18			0.751			
SRERH19				0.825		
SRERH20				0.888		
SRERH21				0.894		
SRERH22				0.892		
SRERH23				0.904		
SRERH24				0.848		
SRERH25					0.644	
SRERH26					0.704	
SRERH27					0.795	
SRERH28					0.871	
SRERH29					0.840	
SRERH30					0.711	
SRERH31						0.832
SRERH32						0.864
SRERH33						0.854
SRERH34						0.836
SRERH35						0.825
SRERH36						0.872

### Reliability

The total reliability of the scale showed adequate ratios (Cronbach Alpha = 0.897; Omega Index = 0.868). Split-half analysis showed adequate values (Alpha 1 = 0.790; Alpha 2 = 0.855; Spearman–Brown Coefficient = 0.837; Guttman Split-half Coefficient = 0.829). The reliability of the subscales also appeared to be acceptable: SRL (Alpha = 0.901; Omega = 0.888); SNL (Alpha = 0.785; Omega = 0.743); SDL (Alpha = 0.873; Omega = 0.852); ERL (Alpha = 0.950; Omega = 0.934); ENL (Alpha = 0.805; Omega = 0.794); EDL (Alpha = 0.939; Omega = 0.914).

### External validity: Psychological well-being

*Formation of groups.* The ANOVA carried out to form the groups showed a significant principal Group Factor effect for SRH-ERH relative to the total score for SRH-ERH [*F*(4.315)  =  1426.336, *p* < 0.001; *eta*^2^ = 0.948, *power* =  1.00; *post-hoc* = 5 > 4 > 3 > 2 > 1, *p* < 0.001]. Levene’s test of error variance based on the mean showed the adequacy of the groups [*L*(4.315) = 1.848, *p* < 0.119]. See Table 18 for the descriptive statistics.

*Effect of the SRH-ERH group on the level of psychological well-being.* The ANOVA carried out showed a significant principal Group Factor effect for SRH-ERH relative to the total score for psychological well-being. [*F*(4) *=* 22.295, *p* < 0.001; *eta*^2^ = 0.241, *power* = 1.00; *post-hoc* = 4.3 > 4 > 2.1 > 2 > 1, *p* < 0.001]. Levene’s test of error variance based on the mean showed the adequacy of the groups [*L*(4.281) = 1.788, *p* < 0.131]. See Table 19 for the descriptive statistics.

**Table 19:** Descriptive statistics for the groups formed (*n* = 286).

SRH-ERH groups	*n* = 286	Mean	(dt)	Well-being	Self-help	Social relationships	Autonomy	Environment	Growth	Purpose
1. LOW	48	−1.20	(0.184)	4.07	4.19	3.91	3.71	3.70	4.44	4.17
2. ML	68	−0.74	(0.140)	4.18	4.26	4.14	3.99	3.96	4.61	4.37
3. *M*	64	−0.19	(0.143)	4.37	4.24	4.50	4.03	4.02	5.05	4.39
4. MH	66	0.29	(0.148)	4.69	4.66	4.78	4.18	4.51	5.24	4.79
5. HIGH	40	0.71	(0.145)	5.14	5.26	5.47	4.43	4.87	5.58	5.24

L, Low; ML, Medium-Low; M, Medium; MH, Medium-High; High, High; 5,4 > 3 > 2,1, *p* < 0.001 in well-being and all components.

### Study 5. Self-regulatory vs. external regulatory inventory in *technology psychology* contexts

#### Descriptive results

The descriptive values found met the normality requirements to be expected of this type of sample. See Table 20.

**Table 20:** Descriptive values for the validation sample (*n* = 760).

Variable	Range	Mean (dt)	Deviation error	Asymmetry		Dev. error kurtosis	Dev. error
SRT	1–5	3.94 (0.767)	0.039	−0.645	0.125	0.314	0.250
NRT	1–5	2.82 (0.726)	0.037	0.197	0.125	0.020	0.250
DRT	1–5	2.67 (0.930)	0.045	0.134	0.125	−0.510	0.249
ERT	1–5	3.72 (0.929)	0.048	−0.573	0.127	0.034	0.254
ENRT	1–5	2.70 (0.923)	0.049	0.206	0.126	−0.388	0.251
EDRT	1–5	2.56 (1.02)	0.053	0.192	0.127	−0.703	0.254

SRT, Self-Regulation in Technology; NRT, Non-Regulation in Technology; DRT, Dys-Regulation in Technology; ERT, External Regulation in Technology; ENRT, External Non-Regulation in Technology; EDRT, External Dys-Regulation in Technology; ^**^p < 0.001; ^*^p < 0.01.

#### Construct validity

##### Correlations

There was a significant negative correlation of SRT with NRT and DRT and a significant positive correlation of NRT with DRT. Across this context, correlations were consistent in direction: negative between ERT and ENRT and positive between ENT and EDRT. Note also the consistent negative and positive correlation of components of the scale with the SR and SR-ERT constructs. See Table 21.

**Table 21:** Correlation between the types of internal and external regulation in Technology Psychology (*n* = 760).

	SRT	NRT	DRT	ERT	ENT	EDT
SRT						
NRT	−0.129^**^					
DRT	−0.160^**^	0.537^**^				
ERT			0.191^**^			
ENT		0.582^**^	0.576^**^	−0.108^*^		
EDT		0.547^**^	0.733^**^	0.190^**^	0.610^**^	
SR	0.214^**^	−0.190^**^	−0.65	0.140^**^	−0.105^*^	0.007
SRT-ERT	0.354^**^	−0.574^**^	−0.692^**^	0.365^**^	0.819^**^	−0.725^**^

SRT, Self-regulation in Technology; NRH, Non-Regulation in Technology; DRH, Dys-Regulation in Technology; ^**^p < 0.001; ^*^p < 0.01.

*Exploratory Factorial Analysis* (EFA). This analysis was carried out with 50% of the sample, obtaining adjusted values: Kaiser–Meyer–Olkin = 0.852; Bartlett’s Sphericity Test (630) = 3,672,012 *p* < 0.001; factor communality was between 0.476 (item 9) and 0.843 (item 3). In the varimax rotation, six factors appeared that explained 68.75% of the variance: Factor 1, *ERT* (14.30% variance) = items 23, 21, 20, 24, 22, 19; Factor 2, *EDRT* (12.58% variance) = 34, 33, 35, 31, 36, 32; Factor 3, *SRT* (10.52%) = 3, 1, 2 4,5,6; Factor 4, *DR* (12.27% variance) = 16, 17,14, 15, 13, 18; Factor 5, ENRT (10.20% variance) = 28, 29, 25, 27, 26, 30; Factor 6, *SNRT* (7.16%) = 7, 11, 12, 9, 8, 10.

*Confirmatory Factorial Structure.* The structural values for this construct appeared to be acceptable [Chi-square = 1628.730, *p* < 0.001; df(702–118) = 584; CH/DF = 2.789; CFI = 0.927; GFI = 0.903; IFI = 0.926; TLI = 0.946; CFI = 0.926, RMSEA = 0.023; RSMR = 0.042; Hoelter = 1,309 (*p* < 0.05), 1,360 (*p* < 0.01)]. See Table 22.

**Table 22:** Standardized total effects (default model; *n* = 380).

	F1	F2	F3	F4	F5	F6
SRERT1	0.763					
SRERT2	0.803					
SRERT3	0.841					
SRERT4	0.810					
SRERT5	0.831					
SRERT6	0.668					
SRERT7		0.545				
SRERT8		0.365				
SRERT9		0.641				
SRERT10		0.550				
SRERT11		0.771				
SRERT12		0.599				
SRERT13			0.647			
SRERT14			0.586			
SRERT15			0.767			
SRERT16			0.741			
SRERT17			0.758			
SRERT18			0.747			
SRERT19				0.757		
SRERT20				0.861		
SRERT21				0.858		
SRERT22				0.868		
SRERT23				0.899		
SRERT24				0.872		
SRERT25					0.638	
SRERT26					0.559	
SRERT27					0.802	
SRERT28					0.855	
SRERT29					0.787	
SRERT30					0.717	
SRERT31						0.712
SRERT32						0.746
SRERT33						0.800
SRERT34						0.847
SRERT35						0.850
SRERT36						0.831

### Reliability

The total reliability of this scale showed adequate values (Cronbach Alpha 0.916; Omega = 0.885). Split-half analysis showed adequate values (Alpha 1 = 0.824; Alpha 2 = 0.882; Spearman–Brown Coefficient = 0.858; Guttman Split-half Coefficient = 0.850). The reliability of the subscales also appeared to be acceptable: SRT (Alpha = 0.881; Omega = 0.876); NRT (Alpha = 0.701; Omega = 0.683); DRT (Alpha = 0.858; Omega = 0.834); ERT (Alpha = 0.943; Omega = 0.925); ENT (Alpha = 0.865; Omega = 0.850); EDT (Alpha = 0.915; Omega = 0.901).

#### External validity: Impatience-hostility (TABP)

*Formation of groups.* The ANOVA carried out to form the groups showed a significant principal Group Factor effect for SRT-ERT relative to the total score for SRT-ERT [*F*(4.294) = 1008.857, *p* < 0.001; *eta*^2^ = 0.932, *power* = 1.00; *post-hoc* = 5 > 4 > 3 > 2 > 1, *p* < 0.001]. Levene’s test of error variance based on the mean showed the adequacy of the groups [*L*(4.296) = 1.749, *p* < 0.128]. See Table 7 for the descriptive statistics.

*Effect of the SRT-ERT group on the level of Type A Behavior Pattern (TABP)*. The ANOVA carried out showed a significant principal effect of the SRT-ERT group relative to the total TABP score [*F*(4.252) = 1.527, *p* < 0.05; *eta*^2^ = 0.035, power = 0.660;], its dimensions [*F*(8.504) = 3.103, *p* < 0.001; *eta*^2^ = 0.064, power = 0.964; *IH*, *F*(4.252) = 4.702, *p* < 0.001; *eta*^2^ = 0.069, power = 1.00; *post-hoc*, 5.4 < 1, 2, *p* < 0.05] and its components [*F*(16,1,008) =2,121, *p* < 0.01; *eta*^2^ = 0.033, power = 0.973; *IMP*, *F*(4,252) = 4.211, *p* < 0.001; *eta*^2^ = 0.063, power = 1.00; *post-hoc*, 5.4 < 1, 2, 3, *p* < 0.05]. Levene’s test of error variance based on the mean showed the adequacy of the groups [*L*(4.225) = 1.788, *p* < 0.199]. See Table 23 for the descriptive statistics.

**Table 23:** Descriptive statistics for the groups formed (*n* = 286).

SRH-ERH groups	*n*	Mean	(dt)	TABP	COW	IH^*^	COMP	OVERW	IMPAC^**^	HOST^*^
1. LOW	46	−1.27	(0.169)	3.58	3.61	3.35	3.51	3.99	3.62	3.09
2. ML	95	−0.84	(0.125)	3.55	3.97	3.28	3.70	4.05	3.57	2.99
3. *M*	81	−0.37	(0.149)	3.35	3.69	3.02	3.54	3.84	3.26	2.94
4. MH	55	0.09	(0.133)	3.34	3.87	2.80	3.50	4.25	3.06	2.78
5. HIGH	24	0.56	(0.189)	3.15	3.61	2.69	3.26	3.95	2.91	2.46

L, Low; ML, Medium-Low; M, Medium; MH, Medium-High; High, High; 5,4 > 3 > 2,1; TABP, Type A Behavior Pattern; COW, Competitiveness-Overwork; IH, Impatience-Hostility; C, Competitiveness; OW, Overwork; I, Impatience; H, Hostility; ^**^p < 0.001; ^*^p < 0.05.

## Discussion

The results obtained provide support for these instruments, and the hypotheses proposed in relation to the instrument presented based on the SR-ER Theory model (de la Fuente, 2017, 2021). *Hypothesis 1*, relating to the demonstration of a stable, valid structure with six components inherent to the theoretical model and common to the different versions of the questionnaire has demonstrated empirical adequacy. It has also been empirically shown that the SR-ER construction allows scores to be ordered in a continuum of the combined scores for the SR-NR-DR (self-regulation) and ER-ENR-EDR (external regulation) components that make up the Scale in its different versions. The reliability and validity results are similar to those found previously with other samples (de la Fuente et al., 2021b; Pachón-Basallo et al., 2021). However, the design of Item 8 of the De-Regulation Scale should be reviewed since it appears to have a lower level of reliability. Future research will allow us to better adapt to the situations of different users in different contexts.

The total score, as an aggregate averaged continuum of Self-Regulation and External Regulation, has allowed the level of regulation in the behavior of a given person to be placed on a conceptual continuum from +1 to −1, as envisaged by the model whereby moving toward +1 represents increasing average regulation and moving toward −1 represents increasing dysregulation. Those scores could be used in practice to assess the degree of personal and contextual regulation of each person in a given environment. Future research should determine the connection between this construct and other more classical constructs in the area of the regulatory difficulties and problems inherent to different pathologies. Some recent studies have suggested that the *dysregulatory* level of subjects is an essential and predictive element in psychiatric pathologies (Billen et al., 2021; Levin-Aspenson et al., 2021); however, those studies have not explicitly addressed the dysregulatory effect of context, which remains to be determined.

Empirical support has also been established for *Hypothesis* 2, that the different versions of the instrument would have adequate construct validity and reliability with sufficient discriminant power or external validity with respect to different constructs of relevance in each field (clinical, educational, health and technology). The same consistent factorial structure with six factors appeared in all versions of the instrument, which can be interpreted as demonstrating factorial invariance (Meredith, 1993).

The relationship between the Self-Regulation, Non-Regulation, and Dys-Regulation constructs was also consistent across the different contexts, giving a stable relationship between Self-Regulation, Non-Regulation, and Dys-Regulation behaviors, both personal (*self-regulated*) and contextual (*externally regulated*). We believe that the ability to distinguish these three types or levels of behavioral regulation is of interest in itself given the behavioral continuum in which they are situated. In addition, we have established that it is possible to externally validate each version of the instrument through a continuous regulation heuristic of *person-context* combinations (with five levels), that has sufficient explanatory power to determine the variability of the different dependent variables analyzed in each context: *clinical* (negative emotional reactivity), *educational* (study achievement emotions), *health* (psychological well-being) and *technology psychology* (impatience-hostility). The consistency found allows us to infer the external convergence validity of the different scales.

A limitation of this work relates to the inconsistency described in the measurement of Item 8, which has now been amended. However, a strength of this work is that the instruments have been translated into other languages. Subsequent research should focus on validation of the instruments with samples from different countries and cultures as a form of transfer of the instrument and the inherent theoretical model and demonstration of factorial invariance, required as part of that process of validation.

## Conclusion

These results support the hypothesis of the types of behavioral regulation—internal and external—proposed by *SR vs. ER Behavior Theory* (de la Fuente, 2021b). As such, they contribute to advance the operational definition of such behavior in the three behavioral level types (SR-NR-DR) and contexts (ER-ENR-EDR). These new constructs and the possibility of measuring them will allow us to detect new behavioral realities and to advance the understanding of the role and effect of personal behavior in its interaction with the environment.

## Implications

The development of these versions of the SR-ER evaluation instrument (de la Fuente, 2020d,e) provides a tool to validate associations between the different levels or types of regulatory behavior, personal and contextual, in different psychological contexts. In addition, it is a step forward in the conceptualization of the typologies of self-regulatory behavior (which can be measured) in relation to other dependent variables measured.

This *new model* and these new *scales* have many academic and professional implications. In the academic sphere, the model will allow the determination of new theoretical and empirical relationships in a continuum of human behavior by confirming the connection between the three levels of self-regulation factors (internal and external). The model will allow the transdiagnostic transition between the three levels of self-regulation proposed from the positive or protective (self-regulation) to the negative or risk level (dysregulation). As such, this analytical framework will help to behaviorally operationalize the p factor in a transdiagnostic way as recently proposed in the field of psychiatry (Kaminski et al., 2022; Smith, 2022). The research agenda for those lines of investigation has recently been laid out as it applies to different fields of Psychology (de la Fuente et al., 2022a). It is also important in the professional arena because the model and the scales allow assessment of the levels of personal and contextual regulation of an individual in a given psychological context. It represents significant progress because it allows contextualization of personal and contextual regulatory factors in interaction (to give a general regulation score). This transcends a purely clinical perspective focused on personality-based factors to explain a given psychopathological behavior. The model also allows assessment and then intervention with knowledge of an individual’s specific behavioral momentum and its development in a particular regulatory direction: SR→NR→DR; SR←NR←DR; ER→ENR→EDR; ER←ENR←EDR.

This development is of theoretical and applied interest, because it supports the use of the concepts of regulation (R), non-regulation (NR), and dys-regulation (DR) which thus far have not been brought together in a coherent theoretical and applied continuum. As such, it opens the door to the exploration of assessment and intervention in different fields:

1. In the professional and academic field of *Clinical Psychology*, the categorization derived from this instrument (SR-NR-DR; ER-ENR-EDR) allows different types of potentially pathological behavior and contexts to be accurately determined. It is assumed that the different levels of self-regulation and external regulation may imply different types of behavioral dysfunction associated with levels of regulation through the *p factor,* as shown by psychiatric research (Billen et al., 2021; Levin-Aspenson et al., 2021).
2. In the professional and academic field of *Educational Psychology*, the existence of these new constructs (SR-NR-DR; ER-ENR-EDR) can help us to understand the factors that regulate the learning processes and the teaching context. Thus, psycho-educational intervention strategies can be based on assessment, evaluation, and intervention in both components of the teaching-learning process.
3. In the professional and academic field of *Health Psychology*, measurement along this continuum (SR-NR-DR; ER-ENR-EDR) will allow us to determine the profiles of individuals who require support and the contexts that promote or do not promote healthful behaviors.
4. In the professional and academic field of the *Psychology of Use of Technology,* measurement along this continuum (SR-NR-DR; ER-ENR-EDR) will allow us to more accurately identify maladjusted behaviors and maladjustive contexts associated with the use of technology at university.

However, there are as-yet unexplored fields to which the theoretical model can be applied, and for which tailored measurement instruments can be developed. Areas of intervention such as Organizational Psychology, Forensic Psychology, Sports Psychology, Psychology of Risk and Catastrophe, Traffic Psychology, and Aviation Psychology could be enriched by these contributions.

## Data availability statement

The raw data supporting the conclusions of this article will be made available by the authors, without undue reservation.

## Ethics statement

This study was reviewed and approved by Comité de Ética de la Investigación, University of Navarra (ref. 2018.170). The patients/participants provided their written informed consent to participate in this study.

## Author contributions

JF and MP-B: R&D Project, idea, design, analysis, and initial writing. JM-V and FP-S: R&D Project, data collection, and revision of the draft. AG-U and PS: review of the final version in English. All authors contributed to the article and approved the submitted version.

## Funding

This research was supported by R&D Project PGC2018-094672-B-I00, University of Navarra, Ministry of Education and Science (Spain), and the European Social Fund (EU); R&D Project UAL18- SEJ-DO31-A-FEDER. University of Almería (Spain), and the European Social Fund (EU) (www.inetas.net).

## Conflict of interest

The authors declare that the research was conducted in the absence of any commercial or financial relationships that could be construed as a potential conflict of interest.

## Publisher’s note

All claims expressed in this article are solely those of the authors and do not necessarily represent those of their affiliated organizations, or those of the publisher, the editors and the reviewers. Any product that may be evaluated in this article, or claim that may be made by its manufacturer, is not guaranteed or endorsed by the publisher.

## Footnotes

^1^ dictionary.apa.org
