# Supplementary material for: Self- vs. External-Regulation Behavior ScaleTM in different psychological contexts: A validation study
Source: Front Psychol. 2022 Oct 26;13:922633. doi: 10.3389/fpsyg.2022.922633 (PMC9644468; doi:10.3389/fpsyg.2022.922633)
Supplement: Supplementary file 1 [file Data_Sheet_1.PDF]

<u>**SELF- vs EXTERNAL REGULATION BEHAVIOR SCALES. (ENGLISH VERSION)**</u>

Jesús Enrique de la Fuente Arias (2022)

17 páginas

<u>**CITAR:**</u>

de la Fuente, J. (2022). *Self- vs External- Regulation Behavior Scales*. Intellectual Property Registry, RPI nº 765-688472 (2022/02/07).

1. **Self- vs External- Regulation Behavior Inventory in Clinical Psychology Context, SR-ER® (de la Fuente, J., 2020c)**

Please, respond to the following questions by ticking the response that best describes how you are.
1 = STRONGLY DISAGREE
2= DISAGREE
3 = UNCERTAIN OR UNSURE
4 = AGREE
5 = STRONGLY AGREE

There are not right or wrong answers. Work quickly and don´t think too long about your answers.

1. I think consciously of my personal needs
Options: 1 2 3 4 5

2. I plan my behavior, setting goals and objectives
Options: 1 2 3 4 5

3. I make decisions in order to achieve positive changes in my behavior
Options: 1 2 3 4 5

4. I observe and keep track of myself in order to see whether I am getting there
Options: 1 2 3 4 5

5. I self-evaluate and reflect on improvements I've made in my behavior
Options: 1 2 3 4 5

6. I learn from mistakes what aspects to improve for future occasions
Options: 1 2 3 4 5

7. I seldom think about my present state and personal needs
Options: 1 2 3 4 5

8. I feel that with time I'll be improving my behavior
Options: 1 2 3 4 5

9. It’s not necessary to make decisions in order to achieve changes in my behavior
Options: 1 2 3 4 5

10. I see behavior changes coming along the way, without giving it too much attention
Options: 1 2 3 4 5

11. I don’t do anything special to produce changes in my behavior, since those will come along on their own

Options: 1 2 3 4 5

12. I sometimes tend to make the same mistakes several times and it looks like I don't learn from experience
Options: 1 2 3 4 5

13. I know just what to do to get around the rules in any situation
Options: 1 2 3 4 5

14. I plan my behavior to get around restrictions, since they seem excessive to me
Options: 1 2 3 4 5

15. I make decisions in order to achieve my personal objectives, at any cost
Options: 1 2 3 4 5

16. I prefer to exercise control for having a good time and enjoying myself, rather than for meeting my obligations
Options: 1 2 3 4 5

17. My self-assessment of my behavior looks mainly at all that I have enjoyed at each moment, nothing else
Options: 1 2 3 4 5

18. It doesn't make sense to change your behavior in life, if that takes away from your enjoyment and satisfaction
Options: 1 2 3 4 5

19.The social context that I live in (family, environment, friends) helps me become aware of my needs for changes in behavior
Options: 1 2 3 4 5

20. The social context that I live in (family, environment, friends) helps me plan my behavior by setting goals and objectives
Options: 1 2 3 4 5

21. The social context that I live in (family, environment, friends) helps me make decisions in order to achieve positive changes in my behavior and personal choices
Options: 1 2 3 4 5

22. The social context that I live in (family, environment, friends) helps me observe and keep track of myself so I can see whether I am achieving what I intended
Options: 1 2 3 4 5

23. The social context that I live in (family, environment, friends) helps me to self-evaluate and reflect on behavior improvements that I have made
Options: 1 2 3 4 5

24. The social context that I live in (family, environment, friends) helps me learn from my mistakes for future occasions and to improve my behavior
Options: 1 2 3 4 5

25. The social context that I live in (family, environment, friends) seldom refers to my behavior or needs for personal improvement
Options: 1 2 3 4 5

26. The social context that I live in (family, environment, friends) feels that I'll be improving my behavior with time. That’s why they don’t interfere much in my life
Options: 1 2 3 4 5

27. The social context that I live in (family, environment, friends) gives me the idea that you don’t need to make specific decisions in order to achieve changes in your behavior. These changes happen by themselves over time
Options: 1 2 3 4 5

28. The social context that I live in (family, environment, friends) lets changes in my behavior come about along the way, without giving it too much attention
Options: 1 2 3 4 5

29. The social context that I live in (family, environment, friends) doesn’t do anything special to produce changes or improvements in my behavior, since those will come along on their own, with time
Options: 1 2 3 4 5

30. The social context that I live in (family, environment, friends) allows me to make the same mistakes several times, even if it looks like I don’t learn from experience
Options: 1 2 3 4 5

31. The social context that I live in (family, environment, friends) encourages me to live in the present and not think too much about my own behavior
Options: 1 2 3 4 5

32. The social context that I live in (family, environment, friends) encourages me to plan behaviors for having fun and enjoying myself, without thinking about limits or restrictions that limit me
Options: 1 2 3 4 5

33. The social context that I live in (family, environment, friends) encourages me to focus on making choices to enjoy the moment, and to postpone my important decisions

Options: 1 2 3 4 5

34. The social context that I live in (family, environment, friends) encourages me to focus my behavioral changes toward living life to the fullest, and to not always be placing limits on myself that keep me from doing what I feel like
Options: 1 2 3 4 5

35. The social context that I live in (family, environment, friends) encourages me to focus on what I have enjoyed in life, when evaluating my own behavior, and not so much on what I have done right by setting ethical or moral limits
Options: 1 2 3 4 5

36. The social context that I live in (family, environment, friends) helps me enjoy myself to the fullest, since it doesn't press me to change my behavior, but rather to do what I feel like, if that makes me happy and live fully
Options: 1 2 3 4 5

2. Self- vs External- Regulation Learning Behavior Inventory in Educational Psychology Context, SRL-ERL® (de la Fuente, J., 2020a)

---

1. I think consciously about my needs related to learning and academic achievement.
Options: 1 2 3 4 5

2. I plan my behavior, setting goals and objectives for learning and achievement.
Options: 1 2 3 4 5

3. I make decisions in order to achieve positive changes in my learning and study behaviors.
Options: 1 2 3 4 5

4. I observe and keep track of myself in order to see whether I am meeting my objectives in learning, study and achievement.
Options: 1 2 3 4 5

5. I self-evaluate and reflect on improvements I've made in my learning, study and achievement behaviors.
Options: 1 2 3 4 5

6. I learn from mistakes what aspects to improve in my learning, study and achievement, for future occasions.
Options: 1 2 3 4 5

7. I seldom think about my present state and needs pertaining to learning and academic achievement.
Options: 1 2 3 4 5

8. I feel that with time I'll be improving my behavior pertaining to learning, study and academic achievement.

Options: 1 2 3 4 5

9. It’s not necessary to make decisions in order to achieve changes in my learning and study behaviors,
Options: 1 2 3 4 5

10. I see changes in my learning and study behaviors coming along the way, without giving it too much attention
Options: 1 2 3 4 5

11. I don’t do anything special to produce changes in my learning and study behavior, since those will come along on their own.
Options: 1 2 3 4 5

12. I sometimes tend to make the same mistakes several times and it looks like I don’t learn from my experience when I underachieve.
Options: 1 2 3 4 5

13. I know just what to do to enjoy myself to the fullest, but not so much for learning, studying and achieving.
Options: 1 2 3 4 5

14. I plan my learning and study behavior to get around restrictions, since they seem excessive to me.
Options: 1 2 3 4 5

15. I make choices for having the most fun, even at the expense of my learning, study and achievement goals.
Options: 1 2 3 4 5

16. I prefer to exercise control for having a good time and enjoying myself, rather than use control for meeting my learning and study obligations.
Options: 1 2 3 4 5

17. My self-assessment of my behavior looks mainly at all that I have enjoyed at each moment, without focusing on mistakes in learning and study.
Options: 1 2 3 4 5

18. It doesn’t make sense in life to change your learning and study behavior, if that takes away from your enjoyment and satisfaction.
Options: 1 2 3 4 5

19. The social context that I live in (family, environment, friends) helps me become aware of my needs in learning, study and achievement.
Options: 1 2 3 4 5

20. The social context that I live in (family, environment, friends) helps me plan my behavior by setting goals and objectives for learning, study and achievement.
Options: 1 2 3 4 5

21. The social context that I live in (family, environment, friends) helps me make decisions in order to achieve positive changes in my learning, study and achievement behaviors.
Options: 1 2 3 4 5

22. The social context that I live in (family, environment, friends) helps me observe and keep track of myself so I can see whether I am meeting my objectives in learning, study and achievement.
Options: 1 2 3 4 5

23. The social context that I live in (family, environment, friends) helps me to self-evaluate and reflect on improvements that I have made in learning, study and achievement behavior.
Options: 1 2 3 4 5

24. The social context that I live in (family, environment, friends) helps me learn from my mistakes for future occasions and to improve my behavior in learning, study and achievement.
Options: 1 2 3 4 5

25. The social context that I live in (family, environment, friends) seldom refers to my behavior or needs for improvement in learning, study and academic achievement.
Options: 1 2 3 4 5

26. The social context that I live in (family, environment, friends) feels that I'll be improving my learning and study behavior with time. That’s why they don’t interfere much in my life.
Options: 1 2 3 4 5

27. The social context that I live in (family, environment, friends) gives me the idea that you don’t need to make specific decisions in order to achieve changes in your learning and study behaviors. These changes happen by themselves over time.
Options: 1 2 3 4 5

28. The social context that I live in (family, environment, friends) lets changes in learning and study behavior come about along the way, without giving it too much attention.
Options: 1 2 3 4 5

29. The social context that I live in (family, environment, friends) doesn’t do anything special to produce changes or improvements in my learning and study behavior, since those will come along on their own, with time.
Options: 1 2 3 4 5

30. The social context that I live in (family, environment, friends) allows me to make the same mistakes in learning, study and achievement several times, even if it looks like I don’t learn from experience.
Options: 1 2 3 4 5

31. The social context that I live in (family, environment, friends) encourages me to live in the present and not think too much about my own behavior in learning, study and achievement. It isn’t that important.
Options: 1 2 3 4 5

32.The social context that I live in (family, environment, friends) encourages me to plan behaviors for having fun and enjoying myself, without thinking about learning and study restrictions that limit me.
Options: 1 2 3 4 5

33. The social context that I live in (family, environment, friends) encourages me to focus on making choices to enjoy the moment, and to postpone learning and study decisions that are important for me.
Options: 1 2 3 4 5

34. The social context that I live in (family, environment, friends) encourages me to focus my behavioral changes toward living life to the fullest, and to not always be placing limits on myself or setting learning and study hours that keep me from doing what I feel like.
Options: 1 2 3 4 5

35. The social context that I live in (family, environment, friends) encourages me to focus on what I have enjoyed in life, when evaluating my own behaviors, and not on what I have done right by setting limits related to learning and study.
Options: 1 2 3 4 5

36. The social context that I live in (family, environment, friends) helps me enjoy myself to the fullest, since it doesn't press me to change my learning and study behavior, but rather to do what I feel like, if that makes me happy and live fully.
Options: 1 2 3 4 5

3. Self- vs External- Regulation Behavior Inventory in Health Psychology Context, SRH-ERH© (de la Fuente, J., 2020b)

Please, respond to the following questions by ticking the response that best describes how you are.
1 = STRONGLY DISAGREE
2= DISAGREE
3 = UNCERTAIN OR UNSURE
4 = AGREE
5 = STRONGLY AGREE

There are not right or wrong answers. Work quickly and don´t think too long about your answers.

1. I think consciously about my health needs
Options: 1 2 3 4 5

2. I plan my health-related behavior by setting goals and objectives
Options: 1 2 3 4 5

3. I make decisions in order to achieve positive changes in my health-related behaviors
Options: 1 2 3 4 5

4. I observe and keep track of myself in order to see whether I am getting there
Options: 1 2 3 4 5

5.I self-evaluate and reflect on improvements I've made in my health-related behavior
Options: 1 2 3 4 5

6. I learn from mistakes what aspects to improve in my health for future occasions
Options: 1 2 3 4 5

7. I seldom think about my state of health and health needs
Options: 1 2 3 4 5

8. I feel that with time I'll be improving my health-related behavior
Options: 1 2 3 4 5

9. It’s not necessary to make decisions in order to achieve changes in my health-related behaviors
Options: 1 2 3 4 5

10. I see health-related changes coming along the way, without giving it too much attention
Options: 1 2 3 4 5

11. I don’t do anything special to produce changes in my health-related behavior, since those will come along on their own
Options: 1 2 3 4 5

12. I sometimes tend to make the same mistakes several times and it looks like I don’t learn from experience when it comes to my health.
Options: 1 2 3 4 5

13. I know just what to do to have a good time, even at the expense of my health
Options: 1 2 3 4 5

14. In my health-related behavior I plan to not follow all the restrictions, since they are excessive
Options: 1 2 3 4 5

15. I make choices in order to achieve changes that harm my health, but I have a good time
Options: 1 2 3 4 5

16. I prefer to exercise control for having a good time and enjoying myself, rather than use control to be healthy
Options: 1 2 3 4 5

17. My self-assessment in health looks at whether I have been enjoying life
Options: 1 2 3 4 5

18. Statement: It doesn’t make sense to change your health-related behavior, if that takes away from your enjoyment and satisfaction
Options: 1 2 3 4 5

19. The social context that I live in (family, environment, friends) helps me become aware of my health-related needs
Options: 1 2 3 4 5

20. The social context that I live in (family, environment, friends) helps me plan my health-related behavior by setting goals and objectives
Options: 1 2 3 4 5

21. The social context that I live in (family, environment, friends) helps me make decisions in order to achieve positive changes in my health-related behaviors
Options: 1 2 3 4 5

22. The social context that I live in (family, environment, friends) helps me observe and keep track of myself so I can see whether I am getting there
Options: 1 2 3 4 5

23. The social context that I live in (family, environment, friends) helps me to self-evaluate and reflect on improvements that I have made in health-related behavior
Options: 1 2 3 4 5

24. The social context that I live in (family, environment, friends) helps me learn from my mistakes for future occasions and to improve my health-related behavior
Options: 1 2 3 4 5

25. The social context that I live in (family, environment, friends) seldom refers to my state of health or health needs
Options: 1 2 3 4 5

26. The social context that I live in (family, environment, friends) feels that I'll be improving my health-related behavior with time. That’s why they don’t interfere in my life
Options: 1 2 3 4 5

27. The social context that I live in (family, environment, friends) gives me the idea that you don’t need to make specific decisions in order to achieve changes in your health-related behaviors
Options: 1 2 3 4 5

28. The social context that I live in (family, environment, friends) lets changes in health-related behavior come about along the way, without giving it too much attention
Options: 1 2 3 4 5

29. The social context that I live in (family, environment, friends) doesn’t do anything special to produce changes in my health-related behavior, since those will come along on their own
Options: 1 2 3 4 5

30. The social context that I live in (family, environment, friends) allows me to make the same mistakes several times, even if it looks like I don’t learn from experience
Options: 1 2 3 4 5

31. The social context that I live in (family, environment, friends) encourages me to live in the present and not think too much about health-related behaviors
Options: 1 2 3 4 5

32. The social context that I live in (family, environment, friends) encourages me to plan behaviors for having fun and enjoying myself, without thinking about health concerns
Options: 1 2 3 4 5

33. The social context that I live in (family, environment, friends) encourages me to focus on making choices to enjoy the moment, and to postpone decisions about my health-related behaviors
Options: 1 2 3 4 5

34. The social context that I live in (family, environment, friends) encourages me to focus any changes in my health-related behaviors toward living life to the fullest, and to not always be placing limits on myself that keep me from doing what I feel like
Options: 1 2 3 4 5

35. The social context that I live in (family, environment, friends) encourages me to focus on what I have enjoyed in life, when evaluating my own health-related behaviors, and not on what I have done right by taking care of my health

Options: 1 2 3 4 5

36. The social context that I live in (family, environment, friends) helps me enjoy myself to the fullest, since it doesn't press me to change my health-related behavior, but rather to do what I feel like, if that makes me happy and live fully
Options: 1 2 3 4 5

4. Self- vs External- Regulation Behavior Inventory in Technological Psychology Context, SRT-ERT ® (de la Fuente, J., 2020d)

This scale is designed to help evaluate the use of technology devices (computer, cell phones, tablets, iPads, smart watches, etc.) in the university class. There are no right or wrong answers.

Please, mark the option that best describes what is true of you or true of your class context, where:

1 = STRONGLY DISAGREE
2= DISAGREE
3 = UNCERTAIN OR UNSURE
4 = AGREE
5 = STRONGLY AGREE

1. I give thought to my own proper use of technology (ICT) in class.
Options: 1 2 3 4 5

2. I plan my technology-related behavior in class.
Options: 1 2 3 4 5

3. I make choices to optimize my technology-related behavior in class.
Options: 1 2 3 4 5

4. I observe and monitor myself to see whether I am making proper use of technologies in class.
Options: 1 2 3 4 5

5. I self-evaluate and reflect on improvements I´ve made in my behavior when using technology in class.
Options: 1 2 3 4 5

6. When I make mistakes I learn what aspects to improve in my ICT use for the future.
Options: 1 2 3 4 5

7. I seldom think about the proper use or abuse of ICT.
Options: 1 2 3 4 5

8. I feel that over time I´ll be getting better in my use of ICT.
Options: 1 2 3 4 5

9. It's not necessary to make choices in order to use ICT in class.
Options: 1 2 3 4 5

10. I improvise changes in my use of ICT on the fly, without giving it too much thought.

Options: 1 2 3 4 5

11. I don’t do anything special to produce behavior changes in my use of ICT, even if I’m not using it right.
Options: 1 2 3 4 5

12. Sometimes I make the same mistakes again and again, and it seems like I don’t learn from experience using ICT.
Options: 1 2 3 4 5

13. I know just what to do to have fun with ICT, even at the expense of my learning and achievement.
Options: 1 2 3 4 5

14. I plan my technology-related behavior to be free of limits, and to use ICT the way I want.
Options: 1 2 3 4 5

15. I make choices so I can use ICT in class the way I want, because no one should tell me how I have to use it.
Options: 1 2 3 4 5

16. I prefer to exercise control so I can have a good time and fully enjoy ICT in class.
Options: 1 2 3 4 5

17. My way of evaluating my use of ICT in class is to look at how I have enjoyed using it.
Options: 1 2 3 4 5

18. It makes no sense to change my behavior in the use of ICT if that keeps me from having a good time during class.
Options: 1 2 3 4 5

19. The class context (university, teachers, classmates) helps me become aware of proper ICT use in class.
Options: 1 2 3 4 5

20. The class context (university, teachers, classmates) helps me plan how to use ICT in class, through goals and objectives.
Options: 1 2 3 4 5

21. The class context (university, teachers, classmates) helps me make choices to ensure proper use of ICT in class.
Options: 1 2 3 4 5

22. The class context (university, teachers, classmates) helps me observe and monitor myself to see whether I am attaining that.
Options: 1 2 3 4 5

23. The class context (university, teachers, classmates) helps me to self-evaluate and reflect on improvements that I’ve made in using ICT.
Options: 1 2 3 4 5

24. The class context (university, teachers, classmates) helps me learn from my mistakes and improve my behavior in the use of ICT.
Options: 1 2 3 4 5

25. In the class context (university, teachers, classmates), proper use of ICT during class is seldom addressed.
Options: 1 2 3 4 5

26.The class context (university, teachers, classmates) considers that over time I will be getting better in ICT use. They don’t interfere in my life.
Options: 1 2 3 4 5

27.The class context (university, teachers, classmates) gives me the idea that you don’t need to make specific choices to achieve changes in the use of ICT during class. Whatever I do is fine.
Options: 1 2 3 4 5

28.The class context (university, teachers, classmates) lets changes in students’ ICT-related behavior come along on their own, without giving too much attention to it.
Options: 1 2 3 4 5

29.The class context (university, teachers, classmates) does not do anything special to make changes in my ICT-related behavior during class.
Options: 1 2 3 4 5

30. The class context (university, teachers, classmates) allows me to repeat the same mistakes, even if it looks like I don’t learn from experience in using ICT.
Options: 1 2 3 4 5

31. The class context (university, teachers, classmates) encourages me to live in the present and not think too much about ICT-related problems.
Options: 1 2 3 4 5

32. The class context (university, teachers, classmates) prompts me to do things for fun and have a good time with ICT during class.
Options: 1 2 3 4 5

33.The class context (university, teachers, classmates) encourages me to focus on making choices to enjoy the moment, and to postpone any choices about the use of ICT in class.
Options: 1 2 3 4 5

34.The class context (university, teachers, classmates) encourages me to focus any behavioral changes in my use of ICT toward living life to the fullest, and to not always be placing limits on myself that keep me from doing what I feel like during class.
Options: 1 2 3 4 5

35. The class context (university, teachers, classmates) influences me to focus only on my enjoyment, nothing else, when I evaluate my use of ICT in class.
Options: 1 2 3 4 5

36. The class context (university, teachers, classmates) helps me enjoy ICT to the fullest, insisting that I do whatever I feel like, if that makes me happy.
Options: 1 2 3 4 5

5. Self- vs External- Regulation Behavior Inventory in Organizational Psychology Context, SRO-ERO® (de la Fuente, J., 2022a)

Please read before start.

Please respond to the following questions by ticking the response that best describes you.
1 = STRONGLY DISAGREE
2 = DISAGREE
3 = UNCERTAIN OR UNSURE
4 = AGREE
5 = STRONGLY AGREE
There are no right or wrong answers. Work quickly and don´t think too long about your answers.

Item No.: 1
Statement: I think consciously of my personal needs in my professional organization.
Options : 1 2 3 4 5

Item No.: 2
Statement: I plan my behavior, setting goals and objectives that fit my professional organization.
Options : 1 2 3 4 5

Item No.: 3
Statement: I make decisions in order to achieve positive changes in my organizational and professional behavior.
Options : 1 2 3 4 5

Item No.: 4
Statement: I observe and keep track of myself to see whether I am meeting my organizational and professional goals.
Options : 1 2 3 4 5

Item No.: 5
Statement: I self-evaluate and reflect on improvements I´ve made in my organizational and professional behavior.
Options : 1 2 3 4 5

Item No.: 6
Statement: I learn from mistakes what aspects to improve in my organizational and professional behavior, on future occasions.
Options : 1 2 3 4 5

Item No.: 7
Statement: I seldom think about my present state and my organizational and professional needs.
Options : 1 2 3 4 5

Item No.: 8
Statement: I feel that, with time, my organizational and professional behavior will improve on its own.
Options : 1 2 3 4 5

Item No.: 9
Statement: It’s not necessary to make decisions in order to achieve changes in my organizational and professional behavior.
Options : 1 2 3 4 5

Item No.: 10
Statement: I manage changes in my organizational and professional behavior on the fly, without giving too much attention to it.
Options : 1 2 3 4 5

Item No.: 11
Statement: I don’t do anything special to make changes in my organizational and professional behavior, since that will come along on its own.
Options : 1 2 3 4 5

Item No.: 12
Statement: Sometimes I make the same organizational and professional mistakes over and over, and it looks like I don’t learn from experience.
Options : 1 2 3 4 5

Item No.: 13
Statement: I know just what to do to get around the rules in any organizational and professional situation.
Options : 1 2 3 4 5

Item No.: 14
Statement: I plan my behavior to get around organizational and professional restrictions, since I consider them excessive.
Options : 1 2 3 4 5

Item No.: 15
Statement: I make decisions so as to achieve my personal, organizational, and professional objectives, at any cost.
Options : 1 2 3 4 5

Item No.: 16
Statement: I prefer to exercise control towards having a good time and enjoying myself, rather than for meeting my organizational and professional obligations.
Options : 1 2 3 4 5

Item No.: 17
Statement: My self-assessment of my behavior looks mainly at everything I have enjoyed at each moment, without other organizational and professional concerns.
Options : 1 2 3 4 5

Item No.: 18
Statement: It doesn’t make sense to change your organizational and professional behavior in life, if that takes away from your enjoyment and satisfaction.
Options : 1 2 3 4 5

Item No.: 19
Statement: The organizational and professional context that I work in (business or job) helps me become aware of my needs for behavioral change in the organization.
Options : 1 2 3 4 5

Item No.: 20
Statement: The organizational and professional context that I work in (business or job) helps me plan my behavior, using goals and objectives related to the organization.
Options : 1 2 3 4 5

Item No.: 21
Statement: The organizational and professional context that I work in (business or job) helps me make decisions in order to achieve positive changes in my behavior and my personal choices.
Options : 1 2 3 4 5

Item No.: 22
Statement: The organizational and professional context that I work in (business or job) helps me observe and keep track of myself so I can see whether I am achieving what I intended.
Options : 1 2 3 4 5

Item No.: 23
Statement: The organizational and professional context that I work in (business or job) helps me to self-evaluate and reflect on behavior improvements that I have made.
Options : 1 2 3 4 5

Item No.: 24
Statement: The organizational and professional context that I work in (business or job) helps me learn from my mistakes for future occasions and to improve my behavior.
Options : 1 2 3 4 5

Item No.: 25
Statement: The organizational and professional context that I work in (business or job) seldom refers to my behavior or needs for personal improvement.
Options : 1 2 3 4 5

Item No.: 26
Statement: The organizational and professional context that I work in (business or job) considers that my behavior will naturally improve over time. So they don't interfere much in my professional decisions.
Options : 1 2 3 4 5

Item No.: 27
Statement: The organizational and professional context that I work in (business or job) gives me the idea that you don't need to make specific decisions to achieve changes in your behavior. These changes happen by themselves, over time.
Options : 1 2 3 4 5

Item No.: 28
Statement: The organizational and professional context that I work in (business or job) allows changes in my behavior to come about along the way, without giving too much attention to it.
Options : 1 2 3 4 5

Item No.: 29
Statement: The organizational and professional context that I work in (business or job) does nothing special to bring about changes or improvements in my organizational behavior, since it is assumed that they will come about on their own, with time.
Options : 1 2 3 4 5

Item No.: 30
Statement: The organizational and professional context that I work in (business or job) allows me to make the same mistakes over and over, even if it looks like I don't learn from experience.
Options : 1 2 3 4 5

Item No.: 31
Statement: The organizational and professional context that I work in (business or job) encourages me to live in the present and not think too much about my own professional and organizational behavior.
Options : 1 2 3 4 5

Item No.: 32
Statement: The organizational and professional context that I work in (business or job) prompts me to plan behaviors for having fun and enjoying myself, without thinking about limits or organizational restrictions that might limit me.
Options : 1 2 3 4 5

Item No.: 33
Statement: The organizational and professional context that I work in (business or job) encourages me to focus on making choices to enjoy the moment, and to postpone professional decisions that are important for me.
Options : 1 2 3 4 5

Item No.: 34
Statement: The organizational and professional context that I work in (business or job) encourages me to focus my behavioral changes toward living life to the fullest, and to not always be placing professional limits on myself that keep me from doing what I feel like.
Options : 1 2 3 4 5

Item No.: 35
Statement: The organizational and professional context that I work in (business or job) encourages me to focus on what I have achieved, when I evaluate my own behavior, and not so much on what I have missed out on by setting ethical or moral limits.
Options : 1 2 3 4 5

Item No.: 36
Statement: The organizational and professional context that I work in (business or job) helps me enjoy myself to the fullest, since it doesn't press me to change my professional behavior, but rather to do what I feel like, if that makes me happy and live fully
Options : 1 2 3 4 5
